# The Association of Type 2 Diabetes Loci Identified in Genome-Wide Association Studies with Metabolic Syndrome and Its Components in a Chinese Population with Type 2 Diabetes

**DOI:** 10.1371/journal.pone.0143607

**Published:** 2015-11-24

**Authors:** Xiaomu Kong, Xuelian Zhang, Xiaoyan Xing, Bo Zhang, Jing Hong, Wenying Yang

**Affiliations:** Department of Endocrinology, China-Japan Friendship Hospital, Beijing, China; Wake Forest School of Medicine, UNITED STATES

## Abstract

Metabolic syndrome (MetS) is prevalent in type 2 diabetes (T2D) patients. The comorbidity of MetS and T2D increases the risk of cardiovascular complications. The aim of the present study was to determine the T2D-related genetic variants that contribute to MetS-related components in T2D patients of Chinese ancestry. We successfully genotyped 25 genome wide association study validated T2D-related single nucleotide polymorphisms (SNPs) among 5,169 T2D individuals and 4,560 normal glycemic controls recruited from the Chinese National Diabetes and Metabolic Disorders Study (DMS). We defined MetS in this population using the harmonized criteria (2009) combined with the Chinese criteria for abdominal obesity. The associations between SNPs and MetS-related components, as well as the associations between SNPs and risk for T2D with or without MetS, were subjected to logistic regression analysis adjusted for age and sex. Results showed that the T2D risk alleles of rs243021 located near *BCL11A*, rs10830963 in *MTNR1B*, and rs2237895 in *KCNQ1* were related to a lower risk for abdominal obesity in T2D patients (rs243021: 0.92 (0.84, 1.00), *P* = 4.42 × 10^−2^; rs10830963: 0.92 (0.85, 1.00), *P* = 4.07 × 10^−2^; rs2237895: 0.89 (0.82, 0.98), *P* = 1.29 × 10^−2^). The T2D risk alleles of rs972283 near *KLF14* contributed to a higher risk of elevated blood pressure (1.10 (1.00, 1.22), *P* = 4.48 × 10^−2^), while the T2D risk allele of rs7903146 in *TCF7L2* was related to a lower risk for elevated blood pressure (0.74 (0.61, 0.90), *P* = 2.56 × 10^−3^). The T2D risk alleles of rs972283 near *KLF14* and rs11634397 near *ZFAND6* were associated with a higher risk for elevated triglycerides (rs972283: 1.11 (1.02, 1.24), *P* = 1.46 × 10^−2^; rs11634397: 1.14 (1.00, 1.29), *P* = 4.66 × 10^−2^), while the T2D risk alleles of rs780094 in *GCKR* and rs7903146 in *TCF7L2* were related to a lower risk of elevated triglycerides (rs780094: 0.86 (0.80, 0.93), *P* = 1.35 × 10^−4^; rs7903146: 0.82 (0.69, 0.98), *P* = 3.18 × 10^−2^). The genotype risk score of the 25 T2D-related SNPs was related to a lower risk for abdominal obesity (*P*_trend_ = 1.29 × 10^−2^) and lower waist circumference (*P* = 2.20 × 10^−3^). Genetic variants of *WFS1*, *CDKAL1*, *CDKN2BAS*, *TCF7L2*, *HHEX*, *KCNQ1*, *TSPAN8/LGR5*, *FTO*, and *TCF2* were associated with the risk for T2D with MetS, as well as the risk for development of T2D with at least one of the MetS components (*P* < 0.05). In addition, genetic variants of *BCL11A*, *GCKR*, *ADAMTS9*, *CDKAL1*, *KLF14*, *CDKN2BAS*, *TCF7L2*, *CDC123/CAMK1D*, *HHEX*, *MTNR1B*, and *KCNQ1* contributed to the risk for T2D without MetS (*P* < 0.05). In conclusion, these findings highlight the contribution of T2D-related genetic loci to MetS in a Chinese Han population. The study also provides insight into the pleotropic effects of genome-wide association loci of diabetes on metabolic regulation.

## Introduction

The pathogenesis of type 2 diabetes (T2D) is mediated by insulin resistance and abnormal insulin secretion. Metabolic syndrome (MetS), which is defined as a cluster of metabolic disturbances related to insulin resistance, including abdominal obesity, hyperglycemia, hypertension, and dyslipidemia, is common in people with T2D. Epidemiologic studies suggested that patients with MetS have a greater risk for cardiovascular disease (CVD) regardless of a previous history of cardiovascular events [1]. Further, concomitant T2D and MetS contribute to a higher prevalence of CVD in T2D population [2]. Therefore, the identification of risk factors for MetS as well as MetS-related components is important for the management and prevention of CVD in T2D.

MetS, along with the associated metabolic traits, is known to be partly hereditary [3,4,5]. For example, in Europe, the heritability of MetS was 23% to 27% in a Dutch isolate [4], and 27% among Italians [3]. It’s shown that MetS has 51% to 60% heritability in Koreans [5]. High-throughput technologies have revealed numerous susceptible genomic loci of both T2D and MetS [6,7,8,9,10,11], mostly in Caucasians. For example, our previous study confirmed the associations between single nucleotide polymorphisms (SNPs) in or near *WFS1*, *CDKAL1*, *CDKN2A/2B*, *CDC123/CAMK1D*, *HHEX*, *TCF7L2*, *KCNQ1*, *MTNR1B* and the risk for T2D in a Chinese Han population as well as the associations between T2D-related SNPs and glycemic traits [12]. On the other hand, candidate genetic approaches found that several T2D-related genetic variants in *TCF7L2* [13,14,15,16], *PPARG* [17,18], and *FTO* [19,20,21] were associated with MetS in the general population. A recently study in Chinese elderly population examined the association between a group of T2D-related genetic variants and MetS, which was not significant [22]. However, finding the genetic contributors of the individual components of MetS is of greater biological interest than the identification of susceptible genomic loci for MetS as a binary trait, since the definition of MetS is based on a constellation of clinical features [9]. In fact, the T2D-related genetic variants showed pleiotropic effects to multiple MetS-related traits other than blood glucose, which included blood pressure, obesity and lipids profiles [23,24,25,26]. Consequently, we speculated that T2D-related genetic variants could contribute substantially to MetS-related components in Chinese population afflicted with T2D.

The aim of the present study was to examine the relationship between genome-wide association study (GWAS)-validated T2D-related genomic loci with the risk for MetS-related components, as well as T2D with MetS and without MetS in a large Chinese Han population comprising of 5,169 T2D patients and 4,560 normal glycemic controls from the Chinese National Diabetes and Metabolic Disorders Study (DMS) [27].

## Methods

### Ethics statement

The study protocol was approved by the Ethics Committee of the China-Japan Friendship Hospital in Beijing and was in accordance with the Helsinki Declaration II. Written informed consents were obtained from all the participants before data collection.

### Study participants and definition of MetS-related components

All the study participants were recruited from the DMS [27]. T2D cases were identified using the 1999 WHO criteria, as fasting plasma glucose (FPG) ≥ 7.0 mmol/l, 2-h oral glucose tolerance test (OGTT) plasma glucose ≥ 11.1 mmol/l, or a self-reported history of T2D. Our analysis included 5,169 T2D patients and 4,560 normal glycemic individuals in DMS, with complete availability of all data pertaining to MetS-related traits.

The diabetic subjects were grouped into 3,651 T2D patients with MetS (MetS T2D) and 1,518 T2D patients without MetS (non-MetS T2D), based on their metabolic phenotypes according to the latest harmonized criteria proposed by the International Diabetes Federation (IDF) and the American Heart Association (AHA) / National Heart, Lung and Blood Institute (NHLBI) in 2009 [28] combined with an amended definition of abdominal obesity for Chinese Hans [29]. MetS was defined as the presence of three or more of the following features: (a) waist circumference (WC) ≥ 90 cm for men or ≥ 85 cm for women [29]; (b) elevated fasting blood glucose ≥ 5.6 mmol/l (100 mg/dl), or drug treatment for hyperglycemia; (c) elevated blood pressure ≥ 130/85 mm Hg, or antihypertensive drug treatment with a history of hypertension; (d) elevated triglycerides (TG) ≥ 1.70 mmol/l (150 mg/dl), or drug treatment for hypertriglyceridemia; and (e) reduced high density lipoprotein-cholesterol (HDL-C) < 1.03 mmol/l (40 mg/dl) for men, or < 1.29 mmol/l (50 mg/dl) for women. In addition, 4,373 out of 4,560 normal glycemic individuals in DMS without MetS were recruited as controls for appropriate comparisons (non-MetS controls), among which 1,956 of them had none of the five MetS components (non-MetS super controls).

### Body measurements and laboratory methods

Body weight, height, and WC were measured using standard methods. Body mass index (BMI) was calculated as weight (kg) divided by squared height (m^2^). The resting blood pressure were measured twice and averaged following standard protocol.

Each participant completed a standard 75 g OGTT after overnight fasting. Blood samples were drawn at 0 min, 30 min, and 2 h after OGTT to measure the plasma glucose and serum insulin concentrations. Serum insulin was measured by double-antibody radioimmunoassay. The homeostasis model assessment for β-cell function (HOMA-B) and insulinogenic index were calculated to estimate β-cell function, and the homeostasis model assessment for insulin resistance (HOMA-IR) and Matsuda index (ISIm) were used to assess insulin resistance using the following formulae:

HOMA-B = fasting serum insulin × 20 / (FPG– 3.5) (with serum insulin in mU/l and plasma glucose in mmol/l) [30]

Insulinogenic index = (serum insulin at 30 min–fasting serum insulin) / (plasma glucose at 30 min–FPG) (with serum insulin in mU/l and plasma glucose in mmol/l) [31]

HOMA-IR = fasting serum insulin × FPG / 22.5 (with serum insulin in mU/l and plasma glucose in mmol/l) [30]

ISIm = 10,000 / (FPG × fasting serum insulin × mean OGTT glucose × mean OGTT insulin)^1/2^ (with serum insulin in mU/l and plasma glucose in mg/dl) [32]

Serum concentrations of fasting TG and HDL-C in plasma were assessed using an automated biochemical analyzer (Olympus, Tokyo, Japan) according to the manufacturer’s instructions.

### Genotyping

Genomic DNA samples were isolated from the peripheral blood using a DNA extraction kit. We selected 31 T2D-related SNPs validated by GWAS previously [33,34,35,36,37,38,39,40,41,42,43,44,45]. Genotyping was accomplished using Illumina GoldenGate Indexing assay (Illumina Inc., San Diego, USA) according to the manufacturer’s instructions. Six of 31 SNPs were excluded because of genotyping success rate lower than 90% (rs231362, rs1531343, rs5945326, and rs13266634) or minor allele frequency (MAF) was less than 0.01 (rs7578597, rs7957197). The overall mean calling rate of remaining 25 SNPs was 97.71%. The concordance rate based on 229 genotyping duplication was 100%. Information of the genotyped SNPs is listed in S1 Table.

### Statistical analysis

The Hardy-Weinberg equilibrium test was tested using a χ^2^ test in the study population (S1 Table). Under an additive genetic assumption, logistic regression analysis was used to test the associations of SNPs with MetS-related components in T2D patients, and was applied in the other analyses as appropriate. Non-Gaussian distributed quantitative traits were natural logarithmically transformed to normal distributions. A linear regression model was used to test the associations between SNPs and quantitative traits. Two multivariable models were tested: model 1, age and sex were adjusted as co-variables; and model 2, age, sex, and BMI were adjusted. A genetic risk score (GRS) of the 25 T2D-related SNPs was constructed using the sum of alleles which were reported as T2D risk alleles in each individual without missing data. The risks for MetS-related components were compared among quartiles of GRS in T2D patients as well as the quantitative traits. Bonferroni correction was used to correct multiple testing. Statistical analyses were performed using SAS (version 9.3; SAS Institute, Cary, NC) and PLINK software (v1.05).

## Results

### Clinical demographics of the study population

The clinical demographics of MetS T2D and non-MetS T2D patients, along with non-MetS controls from DMS, are shown in Table 1. The non-MetS control group and the MetS T2D group included more women than the non-MetS T2D group (*P* < 0.0001). As expected, both T2D groups, regardless of MetS status, manifested significant disorders of adiposity, glucose, blood pressure, and lipids (except for HDL-C) compared with non-MetS control individuals (*P* < 0.0001). Only MetS T2D showed lower fasting HDL-C than controls. Notably, compared with the non-MetS T2D, the MetS T2D group showed significantly increased weight, BMI and WC, elevated glucose and insulin levels during fasting and OGTT, greater insulin resistance and β-cell dysfunction, elevated blood pressure as well as higher concentration of fasting TG, and lower HDL-C levels (all *P* values < 0.0001).

**Table 1:** Clinical demographics of study population. Abbreviations: BMI, body mass index; HDL-C, high density lipoprotein-cholesterol; HOMA-B, the homeostasis model assessment for β-cell function; HOMA-IR, the homeostasis model assessment for insulin resistance; ISIm, Matsuda index; MetS, metabolic syndrome; OGTT, oral glucose tolerance test; T2D, type 2 diabetes. Data are shown as median (interquartile range) or %. All non-Gaussian distributed quantitative traits were natural logarithmically transformed to normalize distributions. *P* values were calculated to assess the intergroup differences using χ^2^ test or one-way ANOVA.

	Non-MetS Control	Non-MetS T2D	MetS T2D	*P*
***n***	4,373	1,518	3,651	
**Male, *n*(%)**	1,393 (31.85)	730 (48.09)	1,506 (41.25)	< 0.0001
**Age, year**	49.00 (44.00, 56.00)	54.00 (45.00, 63.00)^a^	57.00 (48.00, 65.00)^a^ ^b^	< 0.0001
**BMI, kg/m** ^**2**^	22.96 (21.21, 24.67)	23.40 (21.62, 25.03)^a^	26.70 (24.56, 29.03)^a^ ^b^	< 0.0001
**Weight, kg**	59.00 (53.50, 65.00)	60.80 (54.50, 67.00)^a^	69.40 (61.50, 77.20)^a^ ^b^	< 0.0001
**Waist circumference, cm**	78.50 (73.00, 84.00)	81.00 (76.00, 85.00)^a^	91.00 (85.60, 97.00)^a^ ^b^	< 0.0001
**Fasting plasma glucose, mmol/l**	5.00 (4.66, 5.35)	7.16 (5.95, 8.68)^a^	7.41 (6.34, 9.07)^a^ ^b^	< 0.0001
**30-min OGTT glucose, mmol/l**	8.04 (6.93, 9.16)	11.49 (9.48, 13.78)^a^	12.04 (10.00, 14.39)^a^ ^b^	< 0.0001
**120-min OGTT glucose, mmol/l**	5.70 (4.89, 6.55)	12.71 (9.98, 16.60)^a^	13.61 (11.41, 17.00)^a^ ^b^	< 0.0001
**Fasting serum insulin, mU/l**	6.27 (4.88, 8.41)	7.01 (5.09, 9.91)^a^	9.60 (6.75, 13.84)^a^ ^b^	< 0.0001
**30-min OGTT insulin, mU/l**	32.93 (20.90, 52.42)	16.13 (9.22, 31.39)^a^	21.60 (12.71, 37.95)^a^ ^b^	< 0.0001
**120-min OGTT insulin, mU/l**	22.09 (13.71, 34.69)	24.60 (14.05, 43.34)^a^	36.76 (20.76, 66.81)^a^ ^b^	< 0.0001
**Systolic blood pressure, mmHg**	115.00 (107.50,122.50)	120.00 (110.00,130.00)^a^	137.50 (125.00,150.00)^a^ ^b^	< 0.0001
**Diastolic blood pressure, mmHg**	75.00 (70.00, 80.00)	78.00 (70.00, 80.00)^a^	82.50 (78.00, 90.00)^a^ ^b^	< 0.0001
**Triglycerides, mmol/l**	1.01 (0.77, 1.28)	1.13 (0.85, 1.44)^a^	1.96 (1.42, 2.84)^a^ ^b^	< 0.0001
**HDL-C, mmol/l**	1.39 (1.19, 1.62)	1.40 (1.21, 1.61)	1.16 (0.99, 1.36)^a^ ^b^	< 0.0001
**HOMA-IR**	1.38 (1.04, 1.87)	2.29 (1.54, 3.54)^a^	3.27 (2.17, 4.94)^a^ ^b^	< 0.0001
**ISIm**	8.45 (6.28, 11.40)	5.52 (3.81, 7.79)^a^	3.89 (2.63, 5.59)^a^ ^b^	< 0.0001
**HOMA-B, %**	86.30 (61.29,126.64)	40.87 (24.06, 65.67)^a^	49.94 (30.34, 80.16)^a^ ^b^	< 0.0001
**Insulinogenic index**	8.98 (4.53, 16.81)	1.93 (0.50, 4.87)^a^	2.45 (0.82, 5.77)^a^ ^b^	< 0.0001

^a^ *P* value < 0.05 compared with non-MetS control subject in multiple comparison using Dunnett’s test.

^b^ *P* value < 0.05 compared with non-MetS T2D patients in multiple comparison using Dunnett’s test.

### Associations of SNPs with MetS-related components among T2D patients

As shown in Table 2, the reported T2D risk alleles of rs243021 near *BCL11A*, rs10830963 in *MTNR1B*, and rs2237895 in *KCNQ1* were associated with a lower risk for abdominal obesity in T2D patients (rs243021: 0.92 (0.84, 1.00), *P* = 4.42 × 10^−2^; rs10830963: 0.92 (0.85, 1.00), *P* = 4.07 × 10^−2^; rs2237895: 0.89 (0.82, 0.98), *P* = 1.29 × 10^−2^). The T2D risk alleles of rs972283 near *KLF14* contributed to elevated blood pressure (1.10 (1.00, 1.22), *P* = 4.48 × 10^−2^), while the T2D risk allele of rs7903146 in *TCF7L2* was related to a lower risk for elevated blood pressure (0.74 (0.61, 0.90), *P* = 2.56 × 10^−3^). The T2D risk alleles of rs972283 near *KLF14* and rs11634397 near *ZFAND6* were associated with a higher risk for elevated TG level (rs972283: 1.11 (1.02, 1.24), *P* = 1.46 × 10^−2^; rs11634397: 1.14 (1.00, 1.29), *P* = 4.66 × 10^−2^), while the T2D risk alleles of rs780094 in *GCKR* and rs7903146 in *TCF7L2* were related to lower risk for elevated TG (rs780094: 0.86 (0.80, 0.93), *P* = 1.35 × 10^−4^; rs7903146: 0.82 (0.69, 0.98), *P* = 3.18 × 10^−2^). The association between rs780094 and elevated TG remained significant after Bonferroni correction for multiple testing (*P* < 5.00 × 10^−4^). Similar results were achieved when further adjusted for BMI, except that the associations between rs10830963, rs2237895 and abdominal obesity were attenuated (*P* > 0.05). In addition, the associations between single SNPs and the MetS-related components were examined in the entire population of T2D cases and normal glycemic controls with additional adjustment of diabetes status (S2 Table).

**Table 2:** Associations between T2D-related SNPs with MetS-related components in T2D participants. Abbreviations: BMI, body mass index; Chr, chromosome; CI, confidence interval; HDL-C, high density lipoprotein-cholesterol; MetS, metabolic syndrome; OR, odds ratio; SNP, single nucleotide polymorphism; T2D, type 2 diabetes. OR and 95% CI are indicated for the reported T2D risk allele of each SNP with MetS or MetS-related components using logistic regression under an additive assumption using the following models: model 1, age and sex were adjusted as co-variables; and model 2, age, sex, and BMI were adjusted. Associations with *P* value < 0.05 are shown in bold and underlined.

Gene	SNP	Chr.	Major/minor allele^a^		Elevated waist circumference		Elevated blood pressure		Elevated triglycerides		Reduced HDL-C	
					(men: ≥ 90 cm;		(≥ 130/85 mm Hg)		(≥ 1.7 mmol/l)		(men: < 1.03 mmol/l;	
					women: ≥ 85 cm)						women: < 1.29 mmol/l)	
					Model 1	Model 2	Model 1	Model 2	Model 1	Model 2	Model 1	Model 2
*NOTCH2*	rs10923931	1	G/**T**	**OR (95%CI)**	1.03 (0.84,1.27)	1.02 (0.78,1.33)	1.23 (0.97,1.56)	1.24 (0.97,1.59)	0.95 (0.78,1.17)	0.94 (0.76,1.16)	0.93 (0.75,1.15)	0.92 (0.77,1.11)
				***P***	*P* = 7.54×10^−1^	*P* = 8.87×10^−1^	*P* = 9.36×10^−2^	*P* = 8.94×10^−2^	*P* = 6.50×10^−1^	*P* = 5.67×10^−1^	*P* = 5.00×10^−1^	*P* = 4.04×10^−1^
*BCL11A*	rs243021	2	**T**/C	**OR (95%CI)**	0.92 (0.84,1.00)	0.87 (0.78,0.97)	0.98 (0.89,1.09)	0.99 (0.90,1.10)	0.95 (0.88,1.04)	0.96 (0.88,1.04)	0.96 (0.88,1.05)	1.04 (0.99,1.12)
				***P***	*P* = **4.42×10** ^**−2**^	*P* = **1.23×10** ^**−2**^	*P* = 7.63×10^−1^	*P* = 8.96×10^−1^	*P* = 2.59×10^−1^	*P* = 3.21×10^−1^	*P* = 4.27×10^−1^	*P* = 3.75×10^−1^
*GCKR*	rs780094	2	A/**G**	**OR (95%CI)**	0.96 (0.89,1.04)	0.97 (0.88,1.07)	0.92 (0.85,1.01)	0.93 (0.85,1.02)	0.86 (0.80,0.93)	0.86 (0.80,0.94)	1.02 (0.94,1.11)	1.03 (0.95,1.12)
				***P***	*P* = 2.72×10^−1^	*P* = 5.66×10^−1^	*P* = 7.17×10^−2^	*P* = 1.12×10^−1^	*P* = **1.35×10** ^**−4**^	*P* = **3.14×10** ^**−4**^	*P* = 5.60×10^−1^	*P* = 5.07×10^−1^
*PPARG*	rs1801282	3	**C**/G	**OR (95%CI)**	0.94 (0.80,1.11)	1.05 (0.85,1.30)	0.88 (0.73,1.05)	0.92 (0.75,1.11)	0.87 (0.74,1.02)	0.90 (0.76,1.06)	1.04 (0.88,1.23)	0.98 (0.91,1.06)
				***P***	*P* = 4.97×10^−1^	*P* = 6.44×10^−1^	*P* = 1.72×10^−1^	*P* = 3.66×10^−1^	*P* = 9.23×10^−2^	*P* = 2.16×10^−1^	*P* = 6.46×10^−1^	*P* = 7.18×10^−1^
*ADAMTS9*	rs4607103	3	**C**/T	**OR (95%CI)**	0.97 (0.89,1.05)	1.00 (0.90,1.11)	0.93 (0.85,1.02)	0.93 (0.85,1.02)	0.96 (0.88,1.04)	0.97 (0.89,1.05)	1.04 (0.95,1.14)	1.09 (0.99,1.19)
				***P***	*P* = 5.18×10^−1^	*P* = 9.66×10^−1^	*P* = 1.06×10^−1^	*P* = 1.46×10^−1^	*P* = 3.62×10^−1^	*P* = 4.32×10^−1^	*P* = 3.47×10^−1^	*P* = 9.21×10^−2^
*WFS1*	rs10010131	4	**G**/A	**OR (95%CI)**	1.10 (0.90,1.33)	1.20 (0.93,1.56)	1.15 (0.92,1.43)	1.15 (0.92,1.45)	0.96 (0.79,1.16)	0.95 (0.78,1.16)	1.05 (0.85,1.30)	1.02 (0.92,1.12)
				***P***	*P* = 3.47×10^−1^	*P* = 1.69×10^−1^	*P* = 2.24×10^−1^	*P* = 2.27×10^−1^	*P* = 6.67×10^−1^	*P* = 6.27×10^−1^	*P* = 6.17×10^−1^	*P* = 7.28×10^−1^
*ZBED3*	rs4457053	5	A/**G**	**OR (95%CI)**	1.07 (0.89,1.28)	0.95 (0.75,1.20)	1.10 (0.90,1.34)	1.04 (0.84,1.29)	1.03 (0.86,1.23)	1.00 (0.84,1.20)	1.03 (0.86,1.24)	0.96 (0.87,1.05)
				***P***	*P* = 4.59×10^−1^	*P* = 6.73×10^−1^	*P* = 3.71×10^−1^	*P* = 6.97×10^−1^	*P* = 7.24×10^−1^	*P* = 9.75×10^−1^	*P* = 7.33×10^−1^	*P* = 3.49×10^−1^
*CDKAL1*	rs7756992	6	**G**/A	**OR (95%CI)**	0.98 (0.91,1.06)	1.06 (0.96,1.18)	1.01 (0.93,1.10)	1.03 (0.94,1.14)	0.98 (0.91,1.06)	1.00 (0.93,1.09)	0.98 (0.90,1.06)	1.00 (0.90,1.11)
				***P***	*P* = 6.61×10^−1^	*P* = 2.55×10^−1^	*P* = 7.80×10^−1^	*P* = 4.71×10^−1^	*P* = 5.95×10^−1^	*P* = 9.74×10^−1^	*P* = 5.70×10^−1^	*P* = 9.51×10^−1^
*JAZF1*	rs864745	7	**A**/G	**OR (95%CI)**	1.00 (0.92,1.10)	1.01 (0.89,1.14)	0.96 (0.87,1.08)	0.96 (0.86,1.08)	1.05 (0.96,1.15)	1.05 (0.96,1.16)	1.04 (0.95,1.15)	1.01 (0.92,1.10)
				***P***	*P* = 9.60×10^−1^	*P* = 8.91×10^−1^	*P* = 5.05×10^−1^	*P* = 4.84×10^−1^	*P* = 2.63×10^−1^	*P* = 2.59×10^−1^	*P* = 3.61×10^−1^	*P* = 8.96×10^−1^
*KLF14*	rs972283	7	**G**/A	**OR (95%CI)**	1.01 (0.93,1.10)	1.05 (0.94,1.18)	1.10 (1.00,1.22)	1.12 (1.02,1.23)	1.11 (1.02,1.24)	1.12 (1.03,1.23)	0.97 (0.89,1.06)	1.06 (0.89,1.27)
				***P***	*P* = 8.72×10^−1^	*P* = 3.66×10^−1^	*P* = **4.48×10** ^**−2**^	*P* = **2.34×10** ^**−2**^	*P* = **1.46×10** ^**−2**^	*P* = **7.93×10** ^**−3**^	*P* = 5.70×10^−1^	*P* = 4.83×10^−1^
*TP53INP1*	rs896854	8	G/**A**	**OR (95%CI)**	1.06 (0.97,1.15)	1.08 (0.97,1.20)	1.03 (0.94,1.13)	1.01 (0.92,1.12)	1.02 (0.94,1.11)	1.01 (0.93,1.10)	0.99 (0.91,1.08)	0.96 (0.88,1.04)
				***P***	*P* = 1.75×10^−1^	*P* = 1.67×10^−1^	*P* = 5.68×10^−1^	*P* = 7.75×10^−1^	*P* = 6.78×10^−1^	*P* = 8.03×10^−1^	*P* = 8.73×10^−1^	*P* = 3.13×10^−1^
*CDKN2BAS*	rs10811661	9	**T**/C	**OR (95%CI)**	0.93 (0.85,1.00)	1.02 (0.92,1.13)	0.99 (0.90,1.08)	1.03 (0.94,1.14)	1.02 (0.94,1.10)	1.05 (0.97,1.15)	0.96 (0.88,1.04)	0.97 (0.86,1.10)
				***P***	*P* = 6.48×10^−2^	*P* = 6.86×10^−1^	*P* = 7.98×10^−1^	*P* = 4.72×10^−1^	*P* = 6.03×10^−1^	*P* = 1.96×10^−1^	*P* = 3.10×10^−1^	*P* = 6.74×10^−1^
*CHCHD9*	rs13292136	9	**C**/T	**OR (95%CI)**	0.88 (0.77,1.00)	0.81 (0.68,0.96)	1.04 (0.90,1.20)	1.04 (0.89,1.20)	0.95 (0.83,1.09)	0.94 (0.83,1.08)	1.03 (0.89,1.18)	1.01 (0.92,1.10)
				***P***	*P* = 5.23×10^−2^	*P* = **1.62×10** ^**−2**^	*P* = 5.62×10^−1^	*P* = 6.07×10^−1^	*P* = 4.64×10^−1^	*P* = 3.94×10^−1^	*P* = 7.06×10^−1^	*P* = 8.64×10^−1^
*TCF7L2*	rs7903146	10	C/**T**	**OR (95%CI)**	1.04 (0.87,1.25)	1.16 (0.92,1.46)	0.74 (0.61,0.90)	0.75 (0.61,0.92)	0.82 (0.69,0.98)	0.83 (0.69,0.99)	0.92 (0.76,1.11)	0.95 (0.78,1.17)
				***P***	*P* = 6.62×10^−1^	*P* = 2.20×10^−1^	*P* = **2.56×10** ^**−3**^	*P* = **5.03×10** ^**−3**^	*P* = **3.18×10** ^**−2**^	*P* = **4.36×10** ^**−2**^	*P* = 3.64×10^−1^	*P* = 6.52×10^−1^
*CDC123/CAMK1D*	rs12779790	10	A/**G**	**OR (95%CI)**	0.95 (0.86,1.05)	1.02 (0.89,1.16)	0.96 (0.86,1.08)	1.00 (0.89,1.13)	0.99 (0.89,1.09)	1.01 (0.91,1.12)	0.99 (0.89,1.10)	0.93 (0.75,1.15)
				***P***	*P* = 3.43×10^−1^	*P* = 7.81×10^−1^	*P* = 4.78×10^−1^	*P* = 9.82×10^−1^	*P* = 7.82×10^−1^	*P* = 8.59×10^−1^	*P* = 8.29×10^−1^	*P* = 4.79×10^−1^
*HHEX*	rs1111875	10	A/**G**	**OR (95%CI)**	1.05 (0.96,1.15)	1.07 (0.96,1.19)	0.96 (0.87,1.05)	0.95 (0.86,1.05)	1.02 (0.94,1.12)	1.02 (0.94,1.12)	1.00 (0.91,1.09)	1.03 (0.94,1.13)
				***P***	*P* = 2.60×10^−1^	*P* = 2.48×10^−1^	*P* = 3.71×10^−1^	*P* = 3.39×10^−1^	*P* = 5.87×10^−1^	*P* = 6.03×10^−1^	*P* = 9.34×10^−1^	*P* = 4.93×10^−1^
*MTNRIB*	rs10830963	11	C/**G**	**OR (95%CI)**	0.92 (0.85,1.00)	0.94 (0.85,1.04)	1.06 (0.97,1.16)	1.09 (1.00,1.20)	0.96 (0.89,1.04)	0.97 (0.90,1.06)	0.96 (0.88,1.04)	1.02 (0.85,1.23)
				***P***	*P* = **4.07×10** ^**−2**^	*P* = 1.99×10^−1^	*P* = 1.65×10^−1^	*P* = 6.13×10^−2^	*P* = 3.56×10^−1^	*P* = 5.12×10^−1^	*P* = 2.84×10^−1^	*P* = 8.22×10^−1^
*KCNQ1*	rs2237895	11	A/**C**	**OR (95%CI)**	0.89 (0.82,0.98)	0.95 (0.85,1.07)	0.95 (0.86,1.04)	0.97 (0.88,1.08)	1.00 (0.92,1.10)	1.03 (0.94,1.12)	0.91 (0.83,1.00)	1.02 (0.94,1.12)
				***P***	*P* = **1.29×10** ^**−2**^	*P* = 3.77×10^−1^	*P* = 2.61×10^−1^	*P* = 5.76×10^−1^	*P* = 9.53×10^−1^	*P* = 5.84×10^−1^	*P* = 5.32×10^−2^	*P* = 6.09×10^−1^
*CENTD2*	rs1552224	11	**T**/G	**OR (95%CI)**	0.96 (0.83,1.10)	0.99 (0.83,1.19)	1.02 (0.87,1.19)	1.04 (0.88,1.22)	1.08 (0.93,1.23)	1.10 (0.95,1.27)	1.06 (0.93,1.23)	1.01 (0.93,1.11)
				***P***	*P* = 5.55×10^−1^	*P* = 9.26×10^−1^	*P* = 8.19×10^−1^	*P* = 6.24×10^−1^	*P* = 2.87×10^−1^	*P* = 2.03×10^−1^	*P* = 3.78×10^−1^	*P* = 7.65×10^−1^
*TSPAN8/LGR5*	rs7961581	12	T/**C**	**OR (95%CI)**	1.00 (0.91,1.10)	0.96 (0.84,1.08)	0.93 (0.83,1.03)	0.91 (0.82,1.02)	1.04 (0.95,1.15)	1.05 (0.95,1.15)	0.98 (0.89,1.08)	0.98 (0.85,1.12)
				***P***	*P* = 9.63×10^−1^	*P* = 4.67×10^−1^	*P* = 1.50×10^−1^	*P* = 1.12×10^−1^	*P* = 3.84×10^−1^	*P* = 3.73×10^−1^	*P* = 7.22×10^−1^	*P* = 7.23×10^−1^
*ZFAND6*	rs11634397	15	A/**G**	**OR (95%CI)**	1.01 (0.88,1.14)	0.97 (0.82,1.15)	0.95 (0.82,1.09)	0.93 (0.81,1.08)	1.14 (1.00,1.29)	1.14 (1.00,1.30)	0.90 (0.79,1.03)	0.93 (0.81,1.08)
				***P***	*P* = 9.38×10^−1^	*P* = 7.26×10^−1^	*P* = 4.39×10^−1^	*P* = 3.45×10^−1^	*P* = **4.66×10** ^**−2**^	*P* = **4.89×10** ^**−2**^	*P* = 1.16×10^−1^	*P* = 3.50×10^−1^
*PRC1*	rs8042680	15	**A**/C	**OR (95%CI)**	0.83 (0.62,1.11)	0.83 (0.56,1.22)	1.20 (0.88,1.64)	1.30 (0.94,1.82)	1.11 (0.83,1.47)	1.15 (0.85,1.54)	1.22 (0.90,1.64)	1.12 (0.98,1.28)
				***P***	*P* = 2.23×10^−1^	*P* = 3.31×10^−1^	*P* = 2.26×10^−1^	*P* = 1.06×10^−1^	*P* = 4.83×10^−1^	*P* = 3.53×10^−1^	*P* = 2.03×10^−1^	*P* = 9.12×10^−2^
*FTO*	rs8050136	16	C/**A**	**OR (95%CI)**	1.07 (0.95,1.20)	0.96 (0.83,1.12)	1.03 (0.90,1.17)	0.97 (0.85,1.11)	0.95 (0.85,1.07)	0.92 (0.82,1.03)	1.05 (0.93,1.18)	0.81 (0.60,1.10)
				***P***	*P* = 2.68×10^−1^	*P* = 6.21×10^−1^	*P* = 6.98×10^−1^	*P* = 6.79×10^−1^	*P* = 4.13×10^−1^	*P* = 1.49×10^−1^	*P* = 4.51×10^−1^	*P* = 1.76×10^−1^
*FTO*	rs9939609	16	T/**A**	**OR (95%CI)**	1.07 (0.95,1.20)	0.97 (0.83,1.12)	1.03 (0.90,1.17)	0.97 (0.85,1.11)	0.96 (0.86,1.08)	0.93 (0.83,1.04)	1.04 (0.93,1.18)	1.02 (0.91,1.15)
				***P***	*P* = 2.70×10^−1^	*P* = 6.48×10^−1^	*P* = 6.99×10^−1^	*P* = 6.99×10^−1^	*P* = 5.13×10^−1^	*P* = 2.12×10^−1^	*P* = 4.85×10^−1^	*P* = 7.04×10^−1^
*TCF2*	rs7501939	17	C/**T**	**OR (95%CI)**	0.95 (0.87,1.04)	0.98 (0.87,1.09)	1.07 (0.98,1.18)	1.09 (0.99,1.21)	0.97 (0.89,1.06)	0.97 (0.89,1.06)	0.99 (0.91,1.08)	1.03 (0.95,1.12)
				***P***	*P* = 2.63×10^−1^	*P* = 6.80×10^−1^	*P* = 1.42×10^−1^	*P* = 8.09×10^−2^	*P* = 4.83×10^−1^	*P* = 5.51×10^−1^	*P* = 8.33×10^−1^	*P* = 4.66×10^−1^

^a^ Previously reported risk alleles for T2D are shown in bold and underlined.

### GRS of 25 T2D-related SNPs on the risk for MetS-related components among T2D patients

The GRS for 25 T2D-related SNPs showed nominal association with a lower risk for abdominal obesity (WC ≥ 90 cm for men or ≥ 85 cm for women), as well as decreased WC (Table 3). Compared with the lowest quartile of GRS, the ORs (95% CIs) for the risk of abdominal obesity were 0.89 (0.75,1.06), 0.84 (0.71,1.00), and 0.76 (0.63,0.90) for the other three quartiles (*P* for trend = 1.29×10^−2^).Individuals with additional T2D risk alleles showed a decreased WC (Q1~Q4: 89.00 (82.00, 96.00) cm, 88.00 (82.00, 95.00) cm, 88.00 (81.00, 94.00) cm, and 87.00 (80.00, 94.00) cm; *P* = 2.20×10^−3^), and the association remained significant after Bonferroni correction (*P* < 1.25×10^−2^). The associations were no longer significant after additional adjustment for BMI (*P* > 0.05). Further, the GRS did not contribute to the other MetS components in T2D patients (S3 Table). The associations between GRS and MetS-related components were further tested in the entire sample of cases and controls (S4 Table).

**Table 3:** Association between T2D GRS and the risk for abdominal obesity in T2D patients. Abbreviations: BMI, body mass index; CI, confidence interval; GRS, genotype risk score; OR, odds ratio; Q, quartile; T2D, type 2 diabetes; WC, waist circumference. OR and 95% CI are reported for T2D GRS quartiles with the risk for abdominal obesity using logistic regression under an additive assumption using the following models: model 1, age and sex were adjusted as co-variables; model 2, age, sex, and BMI were adjusted. *P* values are calculated for T2D GRS quartiles. *P*_*trend*_ values are calculated for T2D GRS. All non-Gaussian distributed quantitative traits were natural logarithmically transformed to normalize distributions. Associations with *P* values < 0.05 are shown in bold and underlined.

Quartile	Elevated WC		WC, cm
	(men: ≥ 90 cm;		
	women: ≥ 85 cm)		
	Model 1	Model 2	
**Q1**	1	1	89.00 (82.00, 96.00)
**Q2**	0.89 (0.75,1.06)	0.99 (0.79,1.24)	88.00 (82.00, 95.00)
	*P* = 1.78×10^−1^	*P* = 9.34×10^−1^	
**Q3**	0.84 (0.71,1.00)	0.97 (0.77,1.21)	88.00 (81.00, 94.00)
	*P* = 5.24×10^−2^	*P* = 7.73×10^−1^	
**Q4**	0.76 (0.63,0.90)	0.86 (0.68,1.08)	87.00 (80.00, 94.00)
	*P* = **2.10×10** ^**−3**^	*P* = 1.99×10^−1^	
	*P*_trend_ = **1.29×10** ^**−2**^	*P*_trend_ = 4.39×10^−1^	*P* ^a^ = **2.20×10** ^**−3**^
			*P* ^b^ = 3.72×10^−1^

^a^, *P* value calculated for T2D GRS using linear regression under an additive assumption adjusted for age and sex.

^b^, *P* value calculated for T2D GRS using linear regression under an additive assumption adjusted for age, sex and BMI.

### Contribution of SNPs to MetS T2D, non-MetS T2D, and T2D with each MetS-related component

As shown in Table 4, the reported T2D risk allele of rs7756992 in *CDKAL1*, rs10811661 near *CDKN2BAS*, rs7903146 in *TCF7L2*, rs1111875 near *HHEX*, and rs2237895 in *KCNQ1* were predisposed to both MetS T2D and non-MetS T2D compared with non-MetS controls. The associations remained significant after Bonferroni correction (*P* < 2.00 × 10^−3^), except for the association between rs1111875 and T2D, and the association between rs7903146 and MetS T2D. In addition, the T allele of rs7903146 increased the risk for non-MetS T2D by 1.47-fold (*P* = 9.88 × 10^−5^), while only increased the risk for MetS T2D by 1.20-fold (*P* = 2.91 × 10^−2^).

**Table 4:** Associations between T2D-related SNPs with non-MetS T2D and MetS T2D compared to non-MetS controls in DMS. Abbreviations: BMI, body mass index; Chr, chromosome; CI, confidence interval; DMS, Chinese National Diabetes and Metabolic Disorders Study; MetS, metabolic syndrome; OR, odds ratio; SNP, single nucleotide polymorphism; T2D, type 2 diabetes. OR and 95% CI are indicated for the reported T2D risk allele of each SNP using logistic regression under an additive assumption using the following models: model 1, age and sex were adjusted as co-variables; and model 2, age, sex, and BMI were adjusted. Associations with *P* values < 0.05 are shown in bold and underlined.

Gene	SNP	Chr.	Major/minor allele^a^		Non-MetS T2D v.s. Non-MetS controls		MetS T2D v.s. Non-MetS controls	
					(1,518:4,373)		(3,651:4,373)	
					Model 1	Model 2	Model 1	Model 2
*NOTCH2*	rs10923931	1	G/**T**	**OR (95%CI)**	0.89 (0.71,1.11)	0.89 (0.71,1.11)	0.91(0.77,1.08)	0.88 (0.71,1.08)
				***P***	*P* = 3.06×10^−1^	*P* = 3.12×10^−1^	*P* = 2.79×10^−1^	*P* = 2.10×10^−1^
*BCL11A*	rs243021	2	**T**/C	**OR (95%CI)**	1.10 (1.01,1.20)	1.10 (1.00,1.20)	1.02 (0.95,1.09)	0.98 (0.90,1.08)
				***P***	*P* = **3.49×10** ^**−2**^	*P* = **4.70×10** ^**−2**^	*P* = 6.23×10^−1^	*P* = 7.20×10^−1^
*GCKR*	rs780094	2	A/**G**	**OR (95%CI)**	1.13 (1.04,1.23)	1.13 (1.04,1.23)	1.03 (0.97,1.10)	1.01 (0.93,1.09)
				***P***	*P* = **3.61×10** ^**−3**^	*P* = **4.96×10** ^**−3**^	*P* = 3.66×10^−1^	*P* = 8.89×10^−1^
*PPARG*	rs1801282	3	**C**/G	**OR (95%CI)**	1.15 (0.96,1.37)	1.16 (0.97,1.39)	1.04 (0.91,1.19)	1.10 (0.93,1.30)
				***P***	*P* = 1.24×10^−1^	*P* = 1.09×10^−1^	*P* = 5.82×10^−1^	*P* = 2.51×10^−1^
*ADAMTS9*	rs4607103	3	**C**/T	**OR (95%CI)**	1.09 (1.00,1.19)	1.10 (1.00,1.19)	1.02 (0.95,1.09)	1.04 (0.95,1.12)
				***P***	*P* = **4.93×10** ^**−2**^	*P* = **4.38×10** ^**−2**^	*P* = 6.60×10^−1^	*P* = 3.91×10^−1^
*WFS1*	rs10010131	4	**G**/A	**OR (95%CI)**	1.14 (0.93,1.41)	1.16 (0.94,1.45)	1.25 (1.06,1.47)	1.33 (1.10,1.64)
				***P***	*P* = 2.21×10^−1^	*P* = 1.46×10^−1^	*P* = **5.96×10** ^**−3**^	*P* = **4.15×10** ^**−3**^
*ZBED3*	rs4457053	5	A/**G**	**OR (95%CI)**	0.86 (0.71,1.06)	0.87 (0.71,1.07)	1.01 (0.87,1.17)	1.00 (0.83,1.20)
				***P***	*P* = 1.58×10^−1^	*P* = 1.83×10^−1^	*P* = 8.88×10^−1^	*P* = 9.72×10^−1^
*CDKAL1*	rs7756992	6	**G**/A	**OR (95%CI)**	1.15 (1.06,1.25)	1.15 (1.05,1.25)	1.12 (1.05,1.20)	1.18 (1.09,1.28)
				***P***	*P* = **1.07×10** ^**−3**^	*P* = **1.14×10** ^**−3**^	*P* = **4.50×10** ^**−4**^	*P* = **6.84×10** ^**−5**^
*JAZF1*	rs864745	7	**A**/G	**OR (95%CI)**	1.01 (0.92,1.12)	1.02 (0.92,1.12)	1.02 (0.94,1.10)	1.05 (0.95,1.15)
				***P***	*P* = 8.03×10^−1^	*P* = 7.22×10^−1^	*P* = 6.90×10^−1^	*P* = 3.10×10^−1^
*KLF14*	rs972283	7	**G**/A	**OR (95%CI)**	0.90 (0.82,0.99)	0.85 (0.81, 0.98)	1.18 (0.96,1.11)	1.06 (0.97,1.16)
				***P***	*P* = **2.59×10** ^**−2**^	*P* = **2.05×10** ^**−2**^	*P* = 3.66×10^−1^	*P* = 2.13×10^−1^
*TP53INP1*	rs896854	8	G/**A**	**OR (95%CI)**	1.02 (0.93,1.12)	1.02 (0.93,1.12)	1.05 (0.98,1.121)	1.04 (0.95,1.13)
				***P***	*P* = 6.63×10^−1^	*P* = 6.46×10^−1^	*P* = 2.05×10^−1^	*P* = 3.96×10^−1^
*CDKN2BAS*	rs10811661	9	**T**/C	**OR (95%CI)**	1.20 (1.11,1.32)	1.20 (1.11,1.32)	1.16 (1.09,1.25)	1.25 (1.15,1.35)
				***P***	*P* = **1.09×10** ^**−5**^	*P* = **1.42×10** ^**−5**^	*P* = **5.62×10** ^**−6**^	*P* = **5.54×10** ^**−8**^
*CHCHD9*	rs13292136	9	**C**/T	**OR (95%CI)**	1.02 (0.88,1.18)	1.01 (0.88,1.18)	0.97 (0.87,1.08)	0.98 (0.85,1.12)
				***P***	*P* = 7.77×10^−1^	*P* = 8.65×10^−1^	*P* = 5.59×10^−1^	*P* = 7.97×10^−1^
*TCF7L2*	rs7903146	10	C/**T**	**OR (95%CI)**	1.47 (1.21,1.78)	1.47 (1.22,1.79)	1.20 (1.02,1.40)	1.19 (0.98,1.45)
				***P***	*P* = **9.88×10** ^**−5**^	*P* = **8.14×10** ^**−5**^	*P* = **2.91×10** ^**−2**^	*P* = 8.69×10^−2^
*CDC123/CAMK1D*	rs12779790	10	A/**G**	**OR (95%CI)**	1.15 (1.03,1.28)	1.15 (1.03,1.28)	1.03 (0.94,1.12)	1.09 (0.98,1.22)
				***P***	*P* = **1.35×10** ^**−2**^	*P* = **1.39×10** ^**−2**^	*P* = 5.22×10^−1^	*P* = 1.07×10^−1^
*HHEX*	rs1111875	10	A/**G**	**OR (95%CI)**	1.13 (1.03,1.24)	1.13 (1.03,1.24)	1.11 (1.03,1.19)	1.11 (1.02,1.22)
				***P***	*P* = **1.21×10** ^**−2**^	*P* = **1.16×10** ^**−2**^	*P* = **5.48×10** ^**−3**^	*P* = **1.82×10** ^**−2**^
*MTNRIB*	rs10830963	11	C/**G**	**OR (95%CI)**	1.11 (1.02,1.21)	1.10 (1.01,1.20)	1.05 (0.98,1.12)	1.06 (0.97,1.14)
				***P***	*P* = **2.07×10** ^**−2**^	*P* = **2.94×10** ^**−2**^	*P* = 1.72×10^−1^	*P* = 1.93×10^−1^
*KCNQ1*	rs2237895	11	A/**C**	**OR (95%CI)**	1.26 (1.15,1.38)	1.25 (1.14,1.37)	1.16 (1.07,1.24)	1.18 (1.08,1.29)
				***P***	*P* = **1.59×10** ^**−6**^	*P* = **3.20×10** ^**−6**^	*P* = **1.04×10** ^**−4**^	*P* = **3.08×10** ^**−4**^
*CENTD2*	rs1552224	11	**T**/G	**OR (95%CI)**	1.08 (0.92,1.25)	1.06 (0.92,1.25)	1.06 (0.95,1.20)	1.06 (0.93,1.22)
				***P***	*P* = 3.69×10^−1^	*P* = 3.87×10^−1^	*P* = 2.54×10^−1^	*P* = 3.78×10^−1^
*TSPAN8/LGR5*	rs7961581	12	T/**C**	**OR (95%CI)**	1.05 (0.95,1.17)	1.04 (0.94,1.16)	1.09 (1.00,1.18)	1.05 (0.96,1.16)
				***P***	*P* = 3.62×10^−1^	*P* = 4.48×10^−1^	*P* = **4.30×10** ^**−2**^	*P* = 2.94×10^−1^
*ZFAND6*	rs11634397	15	A/**G**	**OR (95%CI)**	1.09 (0.95,1.25)	1.10 (0.96,1.26)	1.03 (0.92,1.14)	1.00 (0.87,1.14)
				***P***	*P* = 2.15×10^−1^	*P* = 1.88×10^−1^	*P* = 6.42×10^−1^	*P* = 9.66×10^−1^
*PRC1*	rs8042680	15	**A**/C	**OR (95%CI)**	0.92 (0.68,1.23)	0.91 (0.68,1.23)	1.11 (0.88,1.43)	1.19 (0.88,1.61)
				***P***	*P* = 5.66×10^−1^	*P* = 5.42×10^−1^	*P* = 3.81×10^−1^	*P* = 2.71×10^−1^
*FTO*	rs8050136	16	C/**A**	**OR (95%CI)**	1.11 (0.98,1.26)	1.10 (0.97,1.25)	1.20 (1.08,1.32)	1.08 (0.95,1.22)
				***P***	*P* = 1.03×10^−1^	*P* = 1.49×10^−1^	*P* = **3.95×10** ^**−4**^	*P* = 2.32×10^−1^
*FTO*	rs9939609	16	T/**A**	**OR (95%CI)**	1.13 (0.99,1.28)	1.12 (0.98,1.27)	1.21 (1.10,1.34)	1.09 (0.96,1.23)
				***P***	*P* = 6.25×10^−2^	*P* = 9.65×10^−2^	*P* = **1.42×10** ^**−4**^	*P* = 1.76×10^−1^
*TCF2*	rs7501939	17	C/**T**	**OR (95%CI)**	1.08 (0.99,1.19)	1.08 (0.99,1.19)	1.12 (1.04,1.20)	1.13 (1.03,1.24)
				***P***	*P* = 9.69×10^−2^	*P* = 9.60×10^−2^	*P* = **3.44×10** ^**−3**^	*P* = **7.45×10** ^**−3**^

^a^ Previously reported risk alleles for T2D are shown in bold and underlined.

On the other hand, rs243021 near *BCL11A*, rs780094 in *GCKR*, rs4607103 near *ADAMTS9*, rs972283 near *KLF14*, rs12779790 near *CDC123/CAMK1D*, and rs10830963 in *MTNR1B* were found related to non-MetS T2D (*P* values ranged from 3.61 × 10^−3^ to 4.93 × 10^−2^), while rs10010131 in *WFS1*, rs7961581 in *TSPAN8/LGR5*, rs8050136 and rs9939609 in *FTO*, and rs7501939 in *TCF2* contributed to MetS T2D (*P* values ranged from 1.42 × 10^−4^ to 4.30 × 10^−2^). The association between *FTO* and MetS T2D remained significant after Bonferroni correction (*P* < 2.00 × 10^−3^). When further adjusted for BMI, the associations between rs7903146, rs7961581, rs8050136, rs9939609 and MetS T2D were attenuated (*P* > 0.05). In addition, the reported T2D risk allele of rs972283 was associated with a decreased risk for non-MetS T2D in the present study, while the risk alleles of the other genomic loci displayed similar trend as previously reported. Comparative sensitivity analysis including only the non-MetS super controls without any MetS components as controls, yielded results confirming most of the above findings (S5 Table).

Further, we compared the allele frequencies between T2D patients combined with one of the MetS-related components (abdominal obesity, high blood pressure, high TG, and low HDL-C) and non-MetS super controls (Table 5). Moderate-to-significant associations were observed between the reported risk alleles for T2D of rs10010131 in *WFS1*, rs7756992 in *CDKAL1*, rs10811661 near *CDKN2BAS*, rs2237895 in *KCNQ1*, rs8050136 and rs9939609 in *FTO*, rs7501939 in *TCF2* and the increased risk for the concurrence of T2D and each MetS component (*P* values ranged from 3.42 × 10^−6^ to 5.61 × 10^−2^). In addition, our findings suggested that the T2D risk allele of rs1111875 near *HHEX* only contributed to an increased risk for T2D combined with abdominal obesity (*P* = 2.63 × 10^−2^), and T2D with elevated TG level (*P* = 4.60 × 10^−2^). The T2D risk allele of rs7903146 in *TCF7L2* specifically contributed to an increased risk for T2D combined with abdominal obesity (*P* = 3.77 × 10^−2^) and the T2D risk allele of rs7961581 in *TSPAN8/LGR5* was related to an increased risk for T2D with elevated TG level (*P* = 4.61 × 10^−2^). As expected, these signals showed a large overlap with the associated SNPs of MetS T2D. Further, after additional adjustment for BMI, the associations between rs7903146, rs1111875, rs8050136, rs9939609 and T2D with abdominal obesity, the associations between rs8050136, rs9939609, rs7501939 and T2D with elevated blood pressure or reduced HDL-C levels, and the associations between rs1111875, rs7961581, rs9939609, rs7501939 and T2D with elevated TG level were attenuated (*P* > 0.05). Moreover, the associations between rs7756992, rs10811661, rs2237895 and T2D with each metabolic component remained significant after Bonferroni correction (*P* < 5.00 × 10^−4^).

**Table 5:** Associations between T2D-related SNPs with T2D combined with each MetS-related component compared with non-MetS super controls in DMS. Abbreviations: BMI, body mass index; Chr, chromosome; CI, confidence interval; DMS, Chinese National Diabetes and Metabolic Disorders Study; HDL-C, high density lipoprotein-cholesterol; MetS, metabolic syndrome; OR, odds ratio; SNP, single nucleotide polymorphism; T2D, type 2 diabetes. OR and 95% CI are indicated for the reported T2D risk allele of each SNP using logistic regression under an additive assumption using the following models: model 1, age and sex were adjusted as co-variables; model 2, age, sex, and BMI were adjusted. Associations with *P* values < 0.05 are shown in bold and underlined.

SNP	Gene	Chr.	Major/minor allele^a^		T2D with elevated waist circumference v.s. Non-MetS super controls		T2D with elevated blood pressure v.s. Non-MetS super controls		T2D with elevated triglycerides v.s. Non-MetS super controls		T2D with reduced HDL-C v.s. Non-MetS super controls	
					(2,871:1,956)		(3,549:1,956)		(2,589:1,956)		(2,160:1,956)	
					Model 1	Model 2	Model 1	Model 2	Model 1	Model 2	Model 1	Model 2
*NOTCH2*	rs10923931	1	G/**T**	**OR (95%CI)**	0.94 (0.75,1.17)	0.99 (0.70,1.40)	1.02 (0.82,1.26)	1.06 (0.81,1.39)	0.92 (0.73,1.15)	0.94 (0.70,1.26)	0.90 (0.71,1.14)	0.86 (0.65,1.15)
				***P***	*P* = 5.59×10^−1^	*P* = 9.53×10^−1^	*P* = 8.82×10^−1^	*P* = 6.57×10^−1^	*P* = 4.74×10^−1^	*P* = 6.94×10^−1^	*P* = 3.80×10^−1^	*P* = 3.16×10^−1^
*BCL11A*	rs243021	2	**T**/C	**OR (95%CI)**	1.05 (0.96,1.15)	0.98 (0.85,1.12)	1.09 (0.99,1.19)	1.10 (0.98,1.22)	1.05 (0.96,1.16)	1.03 (0.92,1.16)	1.06 (0.97,1.18)	1.01 (0.90,1.14)
				***P***	*P* = 2.81×10^−1^	*P* = 7.75×10^−1^	*P* = 6.44×10^−2^	*P* = 1.12×10^−1^	*P* = 2.71×10^−1^	*P* = 6.04×10^−1^	*P* = 2.00×10^−1^	*P* = 8.63×10^−1^
*GCKR*	rs780094	2	A/**G**	**OR (95%CI)**	1.02 (0.93,1.11)	1.03 (0.90,1.17)	1.00 (0.92,1.09)	1.00 (0.90,1.11)	0.94 (0.86,1.03)	0.95 (0.85,1.06)	1.03 (0.94,1.12)	1.01 (0.91,1.13)
				***P***	*P* = 7.31×10^−1^	*P* = 6.83×10^−1^	*P* = 9.80×10^−1^	*P* = 9.78×10^−1^	*P* = 1.67×10^−1^	*P* = 3.16×10^−1^	*P* = 5.30×10^−1^	*P* = 8.19×10^−1^
*PPARG*	rs1801282	3	**C**/G	**OR (95%CI)**	1.05 (0.88,1.27)	1.12 (0.85,1.47)	1.02 (0.86,1.20)	1.09 (0.88,1.35)	1.01 (0.85,1.19)	1.02 (0.82,1.27)	1.14 (0.94,1.35)	1.16 (0.93,1.47)
				***P***	*P* = 5.28×10^−1^	*P* = 4.04×10^−1^	*P* = 8.26×10^−1^	*P* = 4.19×10^−1^	*P* = 9.52×10^−1^	*P* = 8.67×10^−1^	*P* = 1.90×10^−1^	*P* = 1.72×10^−1^
*ADAMTS9*	rs4607103	3	**C**/T	**OR (95%CI)**	1.01 (0.92,1.10)	1.01 (0.88,1.16)	0.99 (0.90,1.08)	1.01 (0.91,1.12)	0.98 (0.89,1.08)	0.99 (0.88,1.11)	1.06 (0.96,1.16)	1.08 (0.95,1.20)
				***P***	*P* = 8.49×10^−1^	*P* = 8.30×10^−1^	*P* = 7.92×10^−1^	*P* = 8.47×10^−1^	*P* = 7.05×10^−1^	*P* = 8.65×10^−1^	*P* = 2.13×10^−1^	*P* = 2.43×10^−1^
*WFS1*	rs10010131	4	**G**/A	**OR (95%CI)**	1.35 (1.09,1.67)	1.49 (1.08,2.04)	1.37 (1.11,1.67)	1.56 (1.20,2.00)	1.28 (1.04,1.59)	1.32 (1.01,1.72)	1.35 (1.08,1.67)	1.30 (1.00,1.69)
				***P***	*P* = **5.50×10** ^**−3**^	*P* = **1.63×10** ^**−2**^	*P* = **3.00×10** ^**−3**^	*P* = **8.65×10** ^**−4**^	*P* = **1.79×10** ^**−2**^	*P* = **4.06×10** ^**−2**^	*P* = **9.35×10** ^**−3**^	*P* = 5.28×10^−2^
*ZBED3*	rs4457053	5	A/**G**	**OR (95%CI)**	1.03 (0.84,1.25)	1.19 (0.88,1.62)	0.99 (0.82,1.20)	0.97 (0.76,1.24)	1.00 (0.82,1.23)	0.96 (0.74,1.24)	1.02 (0.83,1.25)	1.00 (0.78,1.29)
				***P***	*P* = 8.06×10^−1^	*P* = 2.63×10^−1^	*P* = 9.21×10^−1^	*P* = 7.97×10^−1^	*P* = 9.68×10^−1^	*P* = 7.45×10^−1^	*P* = 8.36×10^−1^	*P* = 9.96×10^−1^
*CDKAL1*	rs7756992	6	**G**/A	**OR (95%CI)**	1.18 (1.08,1.28)	1.30 (1.14,1.49)	1.20 (1.11,1.32)	1.27 (1.14,1.41)	1.16 (1.08,1.28)	1.25 (1.11,1.39)	1.16 (1.06,1.28)	1.25 (1.12,1.39)
				***P***	*P* = **2.14×10** ^**−4**^	*P* = **8.31×10** ^**−5**^	*P* = **1.18×10** ^**−5**^	*P* = **8.50×10** ^**−6**^	*P* = **5.25×10** ^**−4**^	*P* = **9.71×10** ^**−5**^	*P* = **8.48×10** ^**−4**^	*P* = **7.69×10** ^**−5**^
*JAZF1*	rs864745	7	**A**/G	**OR (95%CI)**	0.99 (0.89,1.09)	0.97 (0.83,1.14)	0.98 (0.88,1.08)	0.98 (0.87,1.11)	1.01 (0.91,1.12)	1.04 (0.92,1.19)	1.00 (0.90,1.11)	1.01 (0.88,1.15)
				***P***	*P* = 7.92×10^−1^	*P* = 7.25×10^−1^	*P* = 6.74×10^−1^	*P* = 7.56×10^−1^	*P* = 8.73×10^−1^	*P* = 5.25×10^−1^	*P* = 9.75×10^−1^	*P* = 8.87×10^−1^
*KLF14*	rs972283	7	**G**/A	**OR (95%CI)**	0.98 (0.89,1.08)	1.02 (0.88,1.19)	1.02 (0.93,1.12)	1.05 (0.93,1.18)	1.02 (0.93,1.12)	1.03 (0.91,1.16)	0.95 (0.86,1.05)	0.95 (0.85,1.08)
				***P***	*P* = 6.71×10^−1^	*P* = 7.53×10^−1^	*P* = 6.00×10^−1^	*P* = 4.17×10^−1^	*P* = 6.41×10^−1^	*P* = 6.75×10^−1^	*P* = 3.12×10^−1^	*P* = 4.45×10^−1^
*TP53INP1*	rs896854	8	G/**A**	**OR (95%CI)**	1.09 (0.99,1.19)	1.08 (0.94,1.25)	1.05 (0.96,1.15)	1.06 (0.95,1.19)	1.06 (0.96,1.16)	1.06 (0.94,1.19)	1.04 (0.95,1.15)	1.05 (0.94,1.18)
				***P***	*P* = 7.92×10^−2^	*P* = 2.67×10^−1^	*P* = 2.68×10^−1^	*P* = 2.78×10^−1^	*P* = 2.44×10^−1^	*P* = 3.39×10^−1^	*P* = 3.94×10^−1^	*P* = 3.92×10^−1^
*CDKN2BAS*	rs10811661	9	**T**/C	**OR (95%CI)**	1.18 (1.08,1.28)	1.35 (1.18,1.54)	1.19 (1.09,1.30)	1.25 (1.12,1.39)	1.22 (1.11,1.33)	1.28 (1.14,1.43)	1.17 (1.08,1.30)	1.23 (1.11,1.39)
				***P***	*P* = **2.40×10** ^**−4**^	*P* = **1.70×10** ^**−5**^	*P* = **6.82×10** ^**−5**^	*P* = **4.69×10** ^**−5**^	*P* = **1.21×10** ^**−5**^	*P* = **2.06×10** ^**−5**^	*P* = **3.56×10** ^**−4**^	*P* = **1.44×10** ^**−4**^
*CHCHD9*	rs13292136	9	**C**/T	**OR (95%CI)**	0.93 (0.81,1.09)	0.85 (0.68,1.05)	1.03 (0.89,1.19)	1.04 (0.87,1.23)	0.99 (0.85,1.15)	1.03 (0.85,1.25)	0.98 (0.84,1.14)	1.01 (0.84,1.22)
				***P***	*P* = 3.88×10^−1^	*P* = 1.40×10^−1^	*P* = 7.15×10^−1^	*P* = 6.76×10^−1^	*P* = 9.17×10^−1^	*P* = 7.55×10^−1^	*P* = 7.93×10^−1^	*P* = 8.97×10^−1^
*TCF7L2*	rs7903146	10	C/**T**	**OR (95%CI)**	1.25 (1.01,1.54)	1.27 (0.93,1.73)	1.12 (0.91,1.38)	1.04 (0.81,1.35)	1.11 (0.89,1.37)	1.04 (0.80,1.37)	1.19 (0.96,1.49)	1.20 (0.92,1.57)
				***P***	*P* = **3.77×10** ^**−2**^	*P* = 1.36×10^−1^	*P* = 2.83×10^−1^	*P* = 7.55×10^−1^	*P* = 3.63×10^−1^	*P* = 7.55×10^−1^	*P* = 1.15×10^−1^	*P* = 1.73×10^−1^
*CDC123/CAMK1D*	rs12779790	10	A/**G**	**OR (95%CI)**	1.02 (0.91,1.15)	1.14 (0.96,1.37)	1.02 (0.91,1.14)	1.10 (0.96,1.27)	1.04 (0.93,1.17)	1.14 (0.98,1.32)	1.04 (0.92,1.17)	1.12 (0.97,1.29)
				***P***	*P* = 7.03×10^−1^	*P* = 1.39×10^−1^	*P* = 7.46×10^−1^	*P* = 1.68×10^−1^	*P* = 5.12×10^−1^	*P* = 8.98×10^−2^	*P* = 5.34×10^−1^	*P* = 1.39×10^−1^
*HHEX*	rs1111875	10	A/**G**	**OR (95%CI)**	1.11 (1.01,1.23)	1.10 (0.95,1.27)	1.07 (0.98,1.18)	1.01 (0.90,1.13)	1.10 (1.00,1.22)	1.06 (0.94,1.20)	1.08 (0.98,1.20)	1.08 (0.95,1.21)
				***P***	*P* = **2.63×10** ^**−2**^	*P* = 1.93×10^−1^	*P* = 1.49×10^−1^	*P* = 8.61×10^−1^	*P* = **4.60×10** ^**−2**^	*P* = 3.16×10^−1^	*P* = 1.20×10^−1^	*P* = 2.34×10^−1^
*MTNRIB*	rs10830963	11	C/**G**	**OR (95%CI)**	1.00 (0.91,1.09)	0.96 (0.84,1.10)	1.07 (0.98,1.16)	1.08 (0.97,1.20)	1.02 (0.94,1.12)	1.02 (0.91,1.14)	1.01 (0.92,1.10)	0.98 (0.88,1.10)
				***P***	*P* = 9.14×10^−1^	*P* = 5.95×10^−1^	*P* = 1.44×10^−1^	*P* = 1.42×10^−1^	*P* = 6.21×10^−1^	*P* = 7.20×10^−1^	*P* = 8.73×10^−1^	*P* = 7.69×10^−1^
*KCNQ1*	rs2237895	11	A/**C**	**OR (95%CI)**	1.20 (1.09,1.32)	1.22 (1.05,1.42)	1.23 (1.11,1.35)	1.26 (1.12,1.42)	1.26 (1.15,1.40)	1.34 (1.19,1.52)	1.19 (1.07,1.31)	1.25 (1.11,1.42)
				***P***	*P* = **2.50×10** ^**−4**^	*P* = **9.52×10** ^**−3**^	*P* = **2.96×10** ^**−5**^	*P* = **1.39×10** ^**−4**^	*P* = **3.42×10** ^**−6**^	*P* = **3.32×10** ^**−6**^	*P* = **1.12×10** ^**−3**^	*P* = **4.01×10** ^**−4**^
*CENTD2*	rs1552224	11	**T**/G	**OR (95%CI)**	1.00 (0.86,1.16)	0.83 (0.66,1.05)	1.02 (0.88,1.18)	1.01 (0.84,1.22)	1.05 (0.91,1.23)	1.03 (0.85,1.25)	1.03 (0.88,1.22)	0.95 (0.79,1.15)
				***P***	*P* = 9.94×10^−1^	*P* = 1.23×10^−1^	*P* = 8.21×10^−1^	*P* = 9.11×10^−1^	*P* = 4.82×10^−1^	*P* = 7.88×10^−1^	*P* = 6.66×10^−1^	*P* = 6.23×10^−1^
*TSPAN8/LGR5*	rs7961581	12	T/**C**	**OR (95%CI)**	1.08 (0.97,1.20)	0.95 (0.81,1.13)	1.06 (0.95,1.18)	1.01 (0.89,1.15)	1.12 (1.00,1.24)	1.08 (0.94,1.24)	1.08 (0.96,1.21)	1.06 (0.92,1.21)
				***P***	*P* = 1.66×10^−1^	*P* = 5.77×10^−1^	*P* = 2.91×10^−1^	*P* = 8.84×10^−1^	*P* = **4.61×10** ^**−2**^	*P* = 2.90×10^−1^	*P* = 1.97×10^−1^	*P* = 4.30×10^−1^
*ZFAND6*	rs11634397	15	A/**G**	**OR (95%CI)**	1.02 (0.89,1.17)	0.96 (0.77,1.19)	1.00 (0.87,1.14)	1.03 (0.87,1.22)	1.09 (0.94,1.25)	1.07 (0.89,1.27)	0.96 (0.83,1.12)	0.91 (0.76,1.09)
				***P***	*P* = 7.81×10^−1^	*P* = 6.98×10^−1^	*P* = 9.43×10^−1^	*P* = 7.26×10^−1^	*P* = 2.54×10^−1^	*P* = 4.88×10^−1^	*P* = 6.04×10^−1^	*P* = 3.04×10^−1^
*PRC1*	rs8042680	15	**A**/C	**OR (95%CI)**	0.88 (0.64,1.20)	0.92 (0.56,1.52)	1.03 (0.75,1.41)	1.22 (0.81,1.82)	0.97 (0.70,1.33)	1.03 (0.67,1.59)	1.19 (0.83,1.69)	1.16 (0.76,1.79)
				***P***	*P* = 4.20×10^−1^	*P* = 7.43×10^−1^	*P* = 8.73×10^−1^	*P* = 3.43×10^−1^	*P* = 8.45×10^−1^	*P* = 8.80×10^−1^	*P* = 3.40×10^−1^	*P* = 4.90×10^−1^
*FTO*	rs8050136	16	C/**A**	**OR (95%CI)**	1.21 (1.06,1.37)	1.00 (0.82,1.22)	1.18 (1.04,1.35)	1.05 (0.89,1.23)	1.14 (1.00,1.30)	1.05 (0.89,1.25)	1.21 (1.06,1.39)	1.09 (0.93,1.29)
				***P***	*P* = **5.16×10** ^**−3**^	*P* = 9.87×10^−1^	*P* = **1.09×10** ^**−2**^	*P* = 5.74×10^−1^	*P* = 5.61×10^−2^	*P* = 5.60×10^−1^	*P* = **5.73×10** ^**−3**^	*P* = 2.93×10^−1^
*FTO*	rs9939609	16	T/**A**	**OR (95%CI)**	1.23 (1.08,1.40)	1.01 (0.83,1.24)	1.20 (1.05,1.36)	1.07 (0.91,1.25)	1.17 (1.02,1.34)	1.08 (0.91,1.28)	1.23 (1.07,1.41)	1.12 (0.94,1.32)
				***P***	*P* = **1.92×10** ^**−3**^	*P* = 8.90×10^−1^	*P* = **6.06×10** ^**−3**^	*P* = 4.38×10^−1^	*P* = **2.19×10** ^**−2**^	*P* = 3.86×10^−1^	*P* = **3.21×10** ^**−3**^	*P* = 2.05×10^−1^
*TCF2*	rs7501939	17	C/**T**	**OR (95%CI)**	1.10 (1.00,1.21)	1.05 (0.91,1.22)	1.13 (1.03,1.24)	1.12 (1.00,1.25)	1.11 (1.00,1.22)	1.10 (0.97,1.24)	1.11 (1.01,1.23)	1.12 (0.99,1.26)
				***P***	*P* = 5.46×10^−2^	*P* = 4.92×10^−1^	*P* = **1.06×10** ^**−2**^	*P* = 5.69×10^−2^	*P* = **4.25×10** ^**−2**^	*P* = 1.42×10^−1^	*P* = **3.85×10** ^**−2**^	*P* = 7.31×10^−2^

^a^ Previously reported risk alleles for T2D are shown in bold and underlined.

## Discussion

MetS is the major risk factor for diabetes mellitus and CVD [1,46,47]. It is prevalent in patients with T2D [1]. Epidemiological evidence suggests that the comorbidity of T2D and MetS increases the risk of CVD and all-cause mortality among T2D patients [2]. The current diagnostic criteria of MetS are based on the harmonized definition (2009) proposed by IDF and AHA/NHLBI, which requires presence of any three of the five components included in the IDF (2005) definition [28]. A previous study also suggested that the optimal cutoff of waist circumference for abdominal obesity in MetS should be 90 cm for men and 85 cm for women of Chinese [29]. In addition, as MetS is a constellation of clinical features, the association between genetic factor and MetS is strongly driven by the varied definitions of MetS. In the present study, we investigated the associations between 25 T2D-related genetic variants and MetS-related components in DMS, according to the recent harmonized criteria (2009) and the Chinese cutoffs for abdominal obesity, instead of MetS as a binary trait. The results revealed that seven genomic loci, including *BCL11A*, *GCKR*, *KLF14*, *TCF7L2*, *MTNR1B*, *KCNQ1*, and *ZFAND6* were significantly associated with MetS-related components in T2D among Chinese. It also indicated that T2D GRS of the 25 SNPs was related to a lower risk for abdominal obesity and decreased WC. Further, we identified the clusters of SNPs predisposed to non-MetS T2D and MetS T2D compared with the non-MetS controls, as well as the SNPs contributed to the risk for T2D with each MetS-related component. To the best of our knowledge, it is the first study focused on the associations of these candidate genes and MetS-related components among a large and national representative T2D population of Chinese Han.

The reported T2D risk alleles of three SNPs, including the T allele of rs243021 near *BCL11A*, the G allele of the intronic rs10830963 in *MTNR1B*, and the C allele of the intronic rs2237895 in *KCNQ1*, contributed to a decreased risk for abdominal obesity in T2D patients. All the genomic loci were found to be associated with T2D and β-cell dysfunction in human [43,48,49]. *BCL11A* functions as a leukemia disease gene. Its relationship with obesity and adiposity indices is unknown. *MTNR1B* encodes the melatonin receptor, which mediates the action of melatonin. *KCNQ1* encodes the potassium voltage gated channel, KQT like subfamily, member 1. Both *MTNR1B* and *KCNQ1* are expressed in pancreatic islets and play an essential role in the regulation of glucose homeostasis [50,51]. The G allele of rs10830963 was reported to be related to lower BMI [52]. The carriers of G allele were also found to be more sensitive to lifestyle intervention-induced reduction of WC in the Look AHEAD study [53]. Our previous study as well as the other studies have indicated that the C allele of rs2237895 was related to decreased BMI and WC in Chinese [54,55,56]. The results of the present study suggest a potential role of T2D-related genomic loci in abdominal obesity. However, the biological mechanism needs further elucidation.

The reported T2D risk allele G of *KLF14* contributed to the elevated blood pressure and TG levels in T2D patients. KLF14 belongs to the Kruppel-like family of transcription factors. A biological study has demonstrated that KLF14 could participate in the metabolism as a transcriptional activator via regulating the gene networks involved in lipid metabolism [57]. GWAS study identified a group of highly correlated SNPs including rs972283 and rs4731702 (r^2^ = 0.967 in Caucasians reported by 1000 Genome project) upstream of *KLF14* gene in association with multiple metabolic traits and T2D in Caucasians [45], which was recently confirmed by a meta-analysis in a global population [58]. Carriers of T2D risk allele of rs4731702 manifested higher fasting insulin, which suggested a role of its residue gene region in insulin resistance. In addition, rs4731702 was reported to be associated with gene expression in subcutaneous adipose tissue biopsies, which suggested that KLF14 was the master trans-regulator of adipose gene expression [59]. The minor allele T of rs4731702 was previously reported to be associated with increased HDL-C and cholesterol in Chinese Hans [60]. Our present study revealed the contribution of G allele of rs972283 to increased risk of high blood pressure and elevated TG levels. In addition, in contrast to previous reports, the present results indicated that the G allele of rs972283 was protective against non-MetS T2D. However, these findings suggested the significance of KLF14 gene in metabolic modulation and require confirmation studies in the future.

The present study also revealed that the reported T2D risk allele T of rs7903146 in *TCF7L2* was related to decreased risk for elevated blood pressure and TG levels among T2D patients, which were unaltered by adjustment for BMI. The *TCF7L2* gene product is one of the T cell factor/lymphoid enhancer-binding factor transcription factors in Wnt / β-catenin signaling pathway. Studies have revealed the residue block of rs7903146 in *TCF7L2* as the strongest susceptible gene region for T2D in Caucasians [34], which is also applicable across different populations [12]. However, due to a relatively lower MAF of rs7903146 in Chinese Hans (0.029 reported by HapMap project) compared with Caucasians (0.308 reported by 1000 Genome project), larger sample sizes are required to achieve enough statistical power in Chinese. Our present study found a protective association of the T2D risk allele T of rs7903146 with blood pressure and TG levels independent of obesity, while contributing to the increased risk for T2D with or without MetS. It is well known that elevated blood pressure and TG levels are risk factors for T2D, and the above findings appear to be contradictory. However, the association between rs7903146 and hyperglycemia was stronger than its relationship with blood pressure or TG levels, suggesting a greater influence on the metabolic status of the general population [13,14,15,16,61,62,63] than in the diabetics. On the other hand, the pleotropic effects of T allele on the factors promoting MetS or preventing MetS could partly explain the negative findings in previous studies [14,61,62,63]. In addition, a study reported that the T allele was the risk factor for incident hypertension [64] or increased TG level in elderly population [22], which was inconsistent with the present findings. However, the inconsistency could be partly explained by the different study design and population, as both the phenotypes were closely connection to blood glucose. The findings should be confirmed in the future studies.

The reported T2D risk allele G of rs780094 in *GCKR* showed a significant association with decreased risk for elevated TG level. *GCKR* encodes the glucokinase regulatory protein, which is a specific inhibitor of glucokinase in both liver and pancreatic islet cells by competitively forming a protein-protein complex with glucokinase with respect to glucose [65]. GWAS studies revealed that the G allele of rs780094 contributed to a higher risk for T2D, with pleiotropic effects on MetS-related traits including lower TG level in Caucasians and Chinese [66,67,68,69,70,71,72,73,74]. Accordingly, we previously confirmed the above findings in the newly diagnosed T2D from DMS [56]. In addition, the results showed that the T2D risk allele also contributed to the risk for non-MetS T2D in Chinese Hans from DMS, but not to the risk for MetS T2D.

The T2D risk allele G of rs11634397 near *ZFAND6* showed a moderate association with the increased risk for elevated TG level. The gene product of *ZFAND6*, the zinc finger AN1-type domain 6, has a functional interaction with tumor necrosis factor receptor-associated factor 2. It plays a role in the negative regulation of nuclear factor kappaB activation, and is a susceptible gene for T2D in Caucasian [45], but its biologic mechanism was still unclear. We previously observed that G allele of rs11634397 was related to lower insulinogenic index in Chinese [12]. The above finding suggested a potential role of *ZFAND6* in lipid metabolism. Additional studies are needed to confirm or refute the findings.

Interestingly, although obesity is a major risk factor of T2D, individuals with a higher T2D GRS had a lower risk for abdominal obesity and a decreased WC among Chinese T2D patients in the present study. The underlying mechanism is not clear. However, the association could be driven by the genetic loci of which the T2D risk allele was related to a decreased risk for abdominal obesity, including *BCL11A*, *MTNR1B*, and *KCNQ1*. In addition, GRS was not associated with the other MetS components in T2D, which might be partly due to the antagonistic role of the T2D risk alleles. The results suggested that the T2D-related genomic loci have different biological roles in metabolic regulation.

Further, we observed that the associated SNPs clusters of MetS T2D or non-MetS T2D were substantially similar. For example, *TCF7L2*, *CDKN2BAS*, *CDKAL1*, *KCNQ1*, and *HHEX* contributed to both non-MetS T2D and MetS T2D. *MTNR1B*, *CDC123/CAMK1D*, *ADAMTS9*, *BCL11A*, *KLF14*, and *GCKR* were associated with non-MetS T2D, while *TSPAN8/LGR5*, *FTO*, *TCF2*, and *WFS1*were associated with MetS T2D. In our previous study, we confirmed the associations between SNPs in or near *WFS1*, *CDKAL1*, *CDKN2A/2B*, *CDC123/CAMK1D*, *HHEX*, *TCF7L2*, *KCNQ1*, *MTNR1B* and the risk for T2D in DMS [12]. These findings suggested that the associations of genomic loci and T2D could be modified by the individual metabolic status. For example, the associations between *TCF7L2*, *TSPAN8/LGR5*, *FTO* and MetS T2D were attenuated when adjusted for BMI, suggesting the role of obesity. The results also showed that SNPs related to MetS T2D were associated with T2D combined with at least one of the metabolic components. The underlying mechanism requires further elucidation.

The present study has several strengths. First, it investigated the associations between genomic loci and MetS-related components among T2D patients of Chinese Han origin, focusing on 25 genome-wide association loci of T2D, and based on a relatively large T2D population. Second, all the study subjects were Han Chinese from DMS who were genetically homogeneous. Third, the study used an optimal Chinese definition of abdominal obesity. However, our study has limitations. Until now, more than 120 genomic loci have been identified to be related to T2D [75]. In the present study, only 25 index SNPs from 24 genomic loci were examined. Thus, additional studies are warranted to reveal the associations between the other genomic loci and MetS components in Chinese. Only one SNP was selected from each genomic locus, which may result in negative findings due to the lack of adequate coverage of the candidate regions. In addition, the MetS group was conducted in T2D patients, which was not generalizable to the common population. However, better understanding of MetS pathogenesis in T2D is important for the prevention of CVD and other diabetes complications among diabetes patients. Further studies are warranted to replicate these findings.

In conclusion, the present study identified that genomic loci of *BCL11A*, *MTNR1B*, *KCNQ1*, *KLF14*, *TCF7L2*, *GCKR*, and *ZFAND6* were associated with MetS-related components among T2D in a large Chinese population. It’s shown that T2D GRS was related to a lower risk for abdominal obesity. In addition, the results revealed the SNP clusters that contributed to MetS T2D, non-MetS T2D, and T2D with each MetS-related component, respectively. The present study may improve our understanding of the comorbidity of MetS and T2D. It also provides insights into the pleotropic effects of T2D-related genomic loci.

## Supporting Information

S1 Table Information of SNPs genotyped in the present study. Abbreviations: CHB, Han Chinese; Chr, chromosome; EU, European; HW-*P*_*Control*_, Hardy-Weinberg equilibrium in controls; HW-*P*_*T2D*_, Hardy-Weinberg equilibrium in T2D patients; MAF, minor allele frequency; SNP, single nucleotide polymorphism; T2D, type 2 diabetes. ^a^ Previously reported risk alleles for T2D are shown in bold and underlined. ^b^ The allele frequencies of the minor allele in the present study. ^c^ Allele frequencies of the minor allele in CHB and CEU populations of 1000 Genome Project. ^d^ The nearest gene is provided if a SNP is located in the intergenic region.
(DOCX)

S2 Table Associations between T2D-related SNPs with MetS-related components in the entire sample of cases and controls. Abbreviations: BMI, body mass index; Chr, chromosome; CI, confidence interval; HDL-C, high density lipoprotein-cholesterol; MetS, metabolic syndrome; OR, odds ratio; SNP, single nucleotide polymorphism; T2D, type 2 diabetes. ^a^ Previously reported risk alleles for T2D are shown in bold and underlined. OR and 95% CI are indicated for the reported T2D risk allele of each SNP with MetS or MetS-related components using logistic regression under an additive assumption using the following models: model 1, age, sex and T2D status were adjusted as co-variables; and model 2, age, sex, T2D status and BMI were adjusted. Associations with *P* value < 0.05 are shown in bold and underlined.
(DOCX)

S3 Table Non-significant associations between T2D GRS and the risk for MetS-related components in T2D patients. Abbreviations: BMI, body mass index; CI, confidence interval; DBP, diastolic blood pressure; HDL-C, high density lipoprotein-cholesterol; GRS, genotype risk score; MetS, metabolic syndrome; OR, odds ratio; Q, quartile; SBP, systolic blood pressure; T2D, type 2 diabetes; TG, triglycerides. OR and 95% CI are reported for T2D GRS quartiles with the risk for MetS components using logistic regression under an additive assumption using the following models: model 1, age and sex were adjusted as co-variables; and model 2, age, sex, and BMI were adjusted. *P* values are calculated for T2D GRS quartiles. *P*_*trend*_ values are calculated for T2D GRS. All non-Gaussian distributed quantitative traits were natural logarithmically transformed to normalize distributions. ^a^, *P* value calculated for T2D GRS using linear regression under an additive assumption adjusted for age and sex. ^b^, *P* value calculated for T2D GRS using linear regression under an additive assumption adjusted for age, sex and BMI. Associations with *P* values <0.05 are shown in bold and underlined.
(DOCX)

S4 Table Associations between T2D GRS and the risk for MetS-related components in the entire sample of cases and controls. Abbreviations: BMI, body mass index; CI, confidence interval; DBP, diastolic blood pressure; HDL-C, high density lipoprotein-cholesterol; GRS, genotype risk score; MetS, metabolic syndrome; OR, odds ratio; Q, quartile; SBP, systolic blood pressure; T2D, type 2 diabetes; TG, triglycerides; WC, waist circumference. OR and 95% CI are reported for T2D GRS quartiles with the risk for MetS components using logistic regression under an additive assumption using the following models: model 1, age, sex, and T2D status were adjusted as co-variables; and model 2, age, sex, T2D status and BMI were adjusted. *P* values are calculated for T2D GRS quartiles. *P*_*trend*_ values are calculated for T2D GRS. All non-Gaussian distributed quantitative traits were natural logarithmically transformed to normalize distributions. ^a^, *P* value calculated for T2D GRS using linear regression under an additive assumption adjusted for age, sex, and T2D status. ^b^, *P* value calculated for T2D GRS using linear regression under an additive assumption adjusted for age, sex, T2D status and BMI. Associations with *P* values <0.05 are shown in bold and underlined.
(DOCX)

S5 Table Associations between SNPs with MetS T2D and non-MetS T2D compared with non-MetS super controls in DMS. Abbreviations: BMI, body mass index; Chr, chromosome; CI, confidence interval; DMS, Chinese National Diabetes and Metabolic Disorders Study; MetS, metabolic syndrome; OR, odds ratio; SNP, single nucleotide polymorphism; T2D, type 2 diabetes. ^a^ Previously reported risk alleles for T2D are shown in bold and underlined. OR and 95% CI are indicated for the reported T2D risk allele of each SNP using logistic regression under an additive assumption using the following models: model 1, age and sex were adjusted as co-variables; and model 2, age, sex, and BMI were adjusted. Associations with *P* values < 0.05 are shown in bold and underlined.
(DOCX)
