# Supplementary material for: The Association of Type 2 Diabetes Loci Identified in Genome-Wide Association Studies with Metabolic Syndrome and Its Components in a Chinese Population with Type 2 Diabetes
Source: PLoS One. 2015 Nov 24;10(11):e0143607. doi: 10.1371/journal.pone.0143607 (PMC4657988; doi:10.1371/journal.pone.0143607)
Supplement: S1 Table — Abbreviations: CHB, Han Chinese; Chr, chromosome; EU, European; HW-P Control, Hardy-Weinberg equilibrium in controls; HW-P T2D, Hardy-Weinberg equilibrium in T2D patients; MAF, minor allele frequency; SNP, single nucleotide polymorphism; T2D, type 2 diabetes. a Previously reported risk alleles for T2D are shown in bold and underlined. b The allele frequencies of the minor allele in the present study. c Allele frequencies of the minor allele in CHB and CEU populations of 1000 Genome Project. d The nearest gene is provided if a SNP is located in the intergenic region. (DOCX) [file pone.0143607.s001.docx]

**S1 Table. Information of SNPs genotyped in the present study.**

| **SNP** | **Chr.** | **Position** | **Major/minor allele^a^** | **Gene** | **Localization** | **HW-*P_Control_*** | **HW-*P_T2D_*** | **MAF** | | | |
| --- | --- | --- | --- | --- | --- | --- | --- | --- | --- | --- | --- |
|  |  | **(Build 38)** |  |  |  |  |  | **Control^b^** | **T2D^b^** | **CEU^c^** | **CHB^c^** |
| rs10923931 | 1 | 119975336 | G/**T** | *NOTCH2* | Intronic variant | 1.000 | 0.226 | 0.041 | 0.036 | 0.058 | 0.042 |
| rs243021 | 2 | 60357684 | **T**/C | *BCL11A^d^* | Intergenic variant | 1.000 | 0.580 | 0.320 | 0.312 | 0.450 | 0.375 |
| rs780094 | 2 | 27518370 | A/**G** | *GCKR* | Intronic variant | 0.464 | 0.867 | 0.475 | 0.491 | 0.400 | 0.400 |
| rs1801282 | 3 | 12351626 | **C**/G | *PPARG* | Coding, non-synonymous | 1.000 | 1.000 | 0.066 | 0.061 | 0.092 | 0.050 |
| rs4607103 | 3 | 64726228 | **C**/T | *ADAMTS9^d^* | Intergenic variant | 0.081 | 0.385 | 0.379 | 0.370 | 0.200 | 0.425 |
| rs10010131 | 4 | 6291188 | **G**/A | *WFS1* | Intronic variant | 0.305 | 0.012 | 0.047 | 0.039 | 0.333 | 0.017 |
| rs4457053 | 5 | 77129124 | A/**G** | *ZBED3^d^* | Intergenic variant | 0.748 | 0.292 | 0.051 | 0.049 | 0.317 | 0.025 |
| rs7756992 | 6 | 20679478 | **G**/A | *CDKAL1* | Intronic variant | 0.088 | 0.843 | 0.479 | 0.451 | 0.250 | 0.483 |
| rs864745 | 7 | 28140937 | **A**/G | *JAZF1* | Intronic variant | 1.000 | 0.642 | 0.239 | 0.238 | 0.500 | 0.242 |
| rs972283 | 7 | 130782095 | **G**/A | *KLF14^d^* | Intergenic variant | 0.326 | 0.677 | 0.280 | 0.280 | 0.458 | 0.333 |
| rs896854 | 8 | 94948283 | G/**A** | *TP53INP1* | Intronic variant | 0.077 | 0.022 | 0.339 | 0.346 | 0.492 | 0.267 |
| rs10811661 | 9 | 22134095 | **T**/C | *CDKN2BAS^d^* | Intergenic variant | 0.831 | 0.269 | 0.477 | 0.441 | 0.258 | 0.442 |
| rs13292136 | 9 | 79337213 | **C**/T | *CHCHD9^d^* | Intergenic variant | 0.722 | 0.873 | 0.094 | 0.097 | 0.058 | 0.083 |
| rs7903146 | 10 | 112998590 | C/**T** | *TCF7L2* | Intronic variant | 0.322 | 0.148 | 0.040 | 0.051 | 0.308 | 0.029^e^ |
| rs12779790 | 10 | 12286011 | A/**G** | *CDC123/CAMK1D^d^* | Intergenic variant | 0.741 | 0.502 | 0.166 | 0.177 | 0.225 | 0.133 |
| rs1111875 | 10 | 92703125 | A/**G** | *HHEX^d^* | Intergenic variant | 1.000 | 0.047 | 0.283 | 0.305 | 0.408 | 0.342 |
| rs10830963 | 11 | 92975544 | C/**G** | *MTNRIB* | Intronic variant | 0.592 | 0.710 | 0.413 | 0.429 | 0.217 | 0.450 |
| rs2237895 | 11 | 2835964 | A/**C** | *KCNQ1* | Intronic variant | 0.338 | 0.001 | 0.322 | 0.358 | 0.358 | 0.300 |
| rs1552224 | 11 | 72722053 | **T**/G | *CENTD2* | Intronic variant | 0.228 | 0.410 | 0.090 | 0.083 | 0.133 | 0.092 |
| rs7961581 | 12 | 71269322 | T/**C** | *TSPAN8/LGR5* | Intronic variant | 0.160 | 0.174 | 0.204 | 0.216 | 0.250 | 0.175 |
| rs11634397 | 15 | 80139880 | A/**G** | *ZFAND6^d^* | Intergenic variant | 0.346 | 0.093 | 0.098 | 0.102 | 0.383 | 0.058 |
| rs8042680 | 15 | 90978107 | **A**/C | *PRC1* | Intronic variant | 1.000 | 0.088 | 0.019 | 0.018 | 0.242 | 0.000 |
| rs8050136 | 16 | 53782363 | C/**A** | *FTO* | Intronic variant | 0.255 | 0.457 | 0.113 | 0.129 | 0.450 | 0.150 |
| rs9939609 | 16 | 53786615 | T/**A** | *FTO* | Intronic variant | 0.359 | 0.386 | 0.112 | 0.130 | 0.450 | 0.150 |
| rs7501939 | 17 | 37741165 | C/**T** | *TCF2* | Intronic variant | 0.72 | 0.39 | 0.26 | 0.28 | 0.425 | 0.292 |

Abbreviations: CHB, Han Chinese; Chr, chromosome; EU, European; HW-*P_Control_*, Hardy-Weinberg equilibrium in controls; HW-*P_T2D_*, Hardy-Weinberg equilibrium in T2D patients; MAF, minor allele frequency; SNP, single nucleotide polymorphism; T2D, type 2 diabetes.

^a^ Previously reported risk alleles for T2D are shown in bold and underlined.

^b^ The allele frequencies of the minor allele in the present study.

^c^ Allele frequencies of the minor allele in CHB and CEU populations of 1000 Genome Project.

^d^ The nearest gene is provided if a SNP is located in the intergenic region.
