# Supplementary material for: The Association of Type 2 Diabetes Loci Identified in Genome-Wide Association Studies with Metabolic Syndrome and Its Components in a Chinese Population with Type 2 Diabetes
Source: PLoS One. 2015 Nov 24;10(11):e0143607. doi: 10.1371/journal.pone.0143607 (PMC4657988; doi:10.1371/journal.pone.0143607)
Supplement: S2 Table — Abbreviations: BMI, body mass index; Chr, chromosome; CI, confidence interval; HDL-C, high density lipoprotein-cholesterol; MetS, metabolic syndrome; OR, odds ratio; SNP, single nucleotide polymorphism; T2D, type 2 diabetes. a Previously reported risk alleles for T2D are shown in bold and underlined. OR and 95% CI are indicated for the reported T2D risk allele of each SNP with MetS or MetS-related components using logistic regression under an additive assumption using the following models: model 1, age, sex and T2D status were adjusted as co-variables; and model 2, age, sex, T2D status and BMI were adjusted. Associations with P value < 0.05 are shown in bold and underlined. (DOCX) [file pone.0143607.s002.docx]

**S2 Table. Associations between T2D-related SNPs with MetS-related components in the entire sample of cases and controls.**

| **SNP** | **Gene** | **Chr.** | **Major/minor allele^a^** |  | **Elevated waist circumference** | | **Elevated blood pressure** | | **Elevated triglycerides** | | **Reduced HDL-C** | |
| --- | --- | --- | --- | --- | --- | --- | --- | --- | --- | --- | --- | --- |
|  |  |  |  |  | **(men: ≥ 90 cm;** | | **(≥ 130/85 mm Hg)** | | **(≥ 1.7 mmol/l)** | | **(men: < 1.03 mmol/l;** | |
|  |  |  |  |  | **women: ≥ 85 cm)** | |  | |  | | **women: < 1.29 mmol/l)** | |
|  |  |  |  |  | **Model 1** | **Model 2** | **Model 1** | **Model 2** | **Model 1** | **Model 2** | **Model 1** | **Model 2** |
| *NOTCH2* | rs10923931 | 1 | G/**T** | **OR (95%CI)** | 0.99 (0.84,1.17) | 0.96 (0.79,1.18) | 1.22 (1.03,1.45) | 1.23 (1.03,1.47) | 0.97 (0.79,1.18) | 0.96 (0.78,1.17) | 0.90 (0.76,1.05) | 0.90 (0.76,1.05) |
|  |  |  |  | ***P*** | *P* = 9.40×10^-1^ | *P* = 7.14×10^-1^ | *P* = **2.22×10^-2^** | *P* = **2.20×10^-2^** | *P* = 7.54×10^-1^ | *P* = 6.73×10^-1^ | *P* = 1.80×10^-1^ | *P* = 1.80×10^-1^ |
| *BCL11A* | rs243021 | 2 | **T**/C | **OR (95%CI)** | 0.96 (0.89,1.03) | 0.93 (0.85,1.00) | 1.04 (0.97,1.12) | 1.04 (0.96,1.12) | 0.96 (0.88,1.04) | 0.96 (0.88,1.04) | 0.98 (0.92,1.04) | 0.97 (0.91,1.04) |
|  |  |  |  | ***P*** | *P* = 2.46×10^-1^ | *P* = 5.73×10^-2^ | *P* = 2.73×10^-1^ | *P* = 2.96×10^-1^ | *P* = 3.02×10^-1^ | *P* = 3.60×10^-1^ | *P* = 4.72×10^-1^ | *P* = 4.58×10^-1^ |
| *GCKR* | rs780094 | 2 | A/**G** | **OR (95%CI)** | 0.99 (0.93,1.06) | 1.00 (0.93,1.08) | 0.92 (0.86,0.98) | 0.91 (0.85,0.98) | 0.86 (0.80,0.93) | 0.86 (0.80,0.93) | 0.99 (0.93,1.06) | 1.00 (0.94,1.06) |
|  |  |  |  | ***P*** | *P* = 8.38×10^-1^ | *P* = 9.63×10^-1^ | *P* = **9.79×10^-3^** | *P* = **1.03×10^-2^** | *P* = **1.12×10^-4^** | *P* = **2.49×10^-4^** | *P* = 8.30×10^-1^ | *P* = 8.93×10^-1^ |
| *PPARG* | rs1801282 | 3 | **C**/G | **OR (95%CI)** | 0.99 (0.87,1.12) | 1.06 (0.90,1.25) | 0.88 (0.78,1.02) | 0.91 (0.79,1.04) | 0.85 (0.73,1.00) | 0.88 (0.75,1.04) | 1.09 (0.96,1.23) | 1.10 (0.97,1.25) |
|  |  |  |  | ***P*** | *P* = 9.02×10^-1^ | *P* = 4.81×10^-1^ | *P* = 8.76×10^-2^ | *P* = 1.79×10^-1^ | *P* = 5.70×10^-2^ | *P* = 1.50×10^-1^ | *P* = 1.92×10^-1^ | *P* = 1.36×10^-1^ |
| *ADAMTS9* | rs4607103 | 3 | **C**/T | **OR (95%CI)** | 0.96 (0.90,1.03) | 0.99 (0.92,1.08) | 0.95 (0.88,1.02) | 0.96 (0.89,1.03) | 0.97 (0.90,1.05) | 0.98 (0.90,1.06) | 1.00 (0.94,1.06) | 1.01 (0.94,1.08) |
|  |  |  |  | ***P*** | *P* = 2.73×10^-1^ | *P* = 8.36×10^-1^ | *P* = 1.56×10^-1^ | *P* = 2.46×10^-1^ | *P* = 5.41×10^-1^ | *P* = 6.39×10^-1^ | *P* = 9.56×10^-1^ | *P* = 8.69×10^-1^ |
| *WFS1* | rs10010131 | 4 | **G**/A | **OR (95%CI)** | 1.04 (0.89,1.22) | 1.10 (0.91,1.33) | 1.12 (0.95,1.33) | 1.14 (0.96,1.35) | 0.97 (0.80,1.18) | 0.96 (0.79,1.18) | 1.06 (0.91,1.23) | 1.06 (0.91,1.23) |
|  |  |  |  | ***P*** | *P* = 5.76×10^-1^ | *P* = 3.16×10^-1^ | *P* = 1.66×10^-1^ | *P* = 1.40×10^-1^ | *P* = 7.67×10^-1^ | *P* = 7.28×10^-1^ | *P* = 4.32×10^-1^ | *P* = 4.25×10^-1^ |
| *ZBED3* | rs4457053 | 5 | A/**G** | **OR (95%CI)** | 1.01 (0.87,1.17) | 0.93 (0.78,1.11) | 1.06 (0.91,1.24) | 1.04 (0.89,1.22) | 1.02 (0.86,1.22) | 0.99 (0.83,1.19) | 1.03 (0.89,1.19) | 1.02 (0.89,1.18) |
|  |  |  |  | ***P*** | *P* = 8.95×10^-1^ | *P* = 4.19×10^-1^ | *P* = 4.39×10^-1^ | *P* = 6.15×10^-1^ | *P* = 8.16×10^-1^ | *P* = 9.38×10^-1^ | *P* = 6.83×10^-1^ | *P* = 7.72×10^-1^ |
| *CDKAL1* | rs7756992 | 6 | **G**/A | **OR (95%CI)** | 1.00 (0.93,1.06) | 1.05 (0.97,1.14) | 1.03 (0.96,1.10) | 1.04 (0.97,1.11) | 0.98 (0.91,1.06) | 1.00 (0.93,1.09) | 1.01 (0.95,1.08) | 1.02 (0.95,1.09) |
|  |  |  |  | ***P*** | *P* = 9.86×10^-1^ | *P* = 1.89×10^-1^ | *P* = 3.67×10^-1^ | *P* = 2.36×10^-1^ | *P* = 6.69×10^-1^ | *P* = 9.62×10^-1^ | *P* = 6.97×10^-1^ | *P* = 5.68×10^-1^ |
| *JAZF1* | rs864745 | 7 | **A**/G | **OR (95%CI)** | 0.96 (0.89,1.03) | 0.96 (0.88,1.05) | 0.96 (0.88,1.04) | 0.96 (0.89,1.05) | 1.04 (0.95,1.14) | 1.04 (0.95,1.15) | 1.04 (0.97,1.12) | 1.04 (0.97,1.12) |
|  |  |  |  | ***P*** | *P* = 2.65×10^-1^ | *P* = 4.39×10^-1^ | *P* = 3.35×10^-1^ | *P* = 4.01×10^-1^ | *P* = 3.68×10^-1^ | *P* = 3.68×10^-1^ | *P* = 2.84×10^-1^ | *P* = 2.70×10^-1^ |
| *KLF14* | rs972283 | 7 | **G**/A | **OR (95%CI)** | 1.01 (0.93,1.08) | 1.01 (0.93,1.10) | 1.08 (1.00,1.16) | 1.08 (1.00,1.16) | 1.11 (1.02,1.20) | 1.12 (1.03,1.22) | 0.99 (0.93,1.06) | 0.99 (0.93,1.06) |
|  |  |  |  | ***P*** | *P* = 8.57×10^-1^ | *P* = 8.32×10^-1^ | *P* = 5.14×10^-2^ | *P* = 5.71×10^-2^ | *P* = **1.56×10^-2^** | *P* = **9.20×10^-3^** | *P* = 8.88×10^-1^ | *P* = 8.74×10^-1^ |
| *TP53INP1* | rs896854 | 8 | G/**A** | **OR (95%CI)** | 1.04 (0.97,1.11) | 1.04 (0.96,1.13) | 1.08 (1.00,1.16) | 1.07 (0.99,1.15) | 1.01 (0.93,1.10) | 1.01 (0.93,1.10) | 0.97 (0.91,1.04) | 0.97 (0.91,1.04) |
|  |  |  |  | ***P*** | *P* = 2.35×10^-1^ | *P* = 3.43×10^-1^ | *P* = **4.61×10^-2^** | *P* = 7.11×10^-2^ | *P* = 7.36×10^-1^ | *P* = 8.52×10^-1^ | *P* = 4.19×10^-1^ | *P* = 3.85×10^-1^ |
| *CDKN2BAS* | rs10811661 | 9 | **T**/C | **OR (95%CI)** | 0.97 (0.91,1.04) | 1.04 (0.96,1.12) | 1.02 (0.95,1.09) | 1.04 (0.97,1.12) | 1.03 (0.95,1.11) | 1.06 (0.98,1.15) | 1.01 (0.94,1.08) | 1.01 (0.95,1.08) |
|  |  |  |  | ***P*** | *P* = 4.17×10^-1^ | *P* = 3.03×10^-1^ | *P* = 5.70×10^-1^ | *P* = 2.20×10^-1^ | *P* = 4.99×10^-1^ | *P* = 1.51×10^-1^ | *P* = 8.56×10^-1^ | *P* = 6.83×10^-1^ |
| *CHCHD9* | rs13292136 | 9 | **C**/T | **OR (95%CI)** | 0.92 (0.82,1.02) | 0.88 (0.77,1.00) | 1.11 (0.99,1.25) | 1.11 (0.99,1.25) | 0.96 (0.84,1.09) | 0.95 (0.83,1.09) | 1.04 (0.93,1.15) | 1.03 (0.93,1.15) |
|  |  |  |  | ***P*** | *P* = 1.06×10^-1^ | *P* = 5.07×10^-2^ | *P* = 7.22×10^-2^ | *P* = 8.37×10^-2^ | *P* = 5.26×10^-1^ | *P* = 4.50×10^-1^ | *P* = 4.89×10^-1^ | *P* = 5.13×10^-1^ |
| *TCF7L2* | rs7903146 | 10 | C/**T** | **OR (95%CI)** | 0.94 (0.81,1.09) | 0.99 (0.82,1.19) | 0.76 (0.65,0.89) | 0.77 (0.65,0.91) | 0.82 (0.69,0.98) | 0.83 (0.69,0.99) | 0.97 (0.84,1.13) | 0.98 (0.84,1.13) |
|  |  |  |  | ***P*** | *P* = 4.21×10^-1^ | *P* = 9.11×10^-1^ | *P* = **8.93×10^-4^** | *P* = **2.00×10^-3^** | *P* = **2.97×10^-2^** | *P* = **4.10×10^-2^** | *P* = 7.02×10^-1^ | *P* = 7.50×10^-1^ |
| *CDC123/CAMK1D* | rs12779790 | 10 | A/**G** | **OR (95%CI)** | 0.97 (0.89,1.05) | 1.03 (0.93,1.14) | 0.96 (0.88,1.05) | 0.98 (0.90,1.08) | 0.99 (0.89,1.09) | 1.01 (0.91,1.12) | 0.99 (0.91,1.07) | 0.99 (0.92,1.08) |
|  |  |  |  | ***P*** | *P* = 4.60×10^-1^ | *P* = 5.83×10^-1^ | *P* = 3.30×10^-1^ | *P* = 7.06×10^-1^ | *P* = 7.72×10^-1^ | *P* = 8.67×10^-1^ | *P* = 7.41×10^-1^ | *P* = 8.82×10^-1^ |
| *HHEX* | rs1111875 | 10 | A/**G** | **OR (95%CI)** | 1.03 (0.96,1.11) | 1.04 (0.96,1.14) | 0.95 (0.88,1.02) | 0.94 (0.87,1.02) | 1.02 (0.93,1.11) | 1.01 (0.93,1.11) | 0.99 (0.92,1.05) | 0.98 (0.92,1.05) |
|  |  |  |  | ***P*** | *P* = 3.96×10^-1^ | *P* = 3.23×10^-1^ | *P* = 1.45×10^-1^ | *P* = 1.34×10^-1^ | *P* = 7.18×10^-1^ | *P* = 7.43×10^-1^ | *P* = 6.68×10^-1^ | *P* = 6.23×10^-1^ |
| *MTNRIB* | rs10830963 | 11 | C/**G** | **OR (95%CI)** | 0.93 (0.87,0.99) | 0.92 (0.85,1.00) | 1.00 (0.93,1.07) | 1.00 (0.94,1.08) | 0.96 (0.89,1.04) | 0.97 (0.90,1.05) | 0.94 (0.89,1.00) | 0.94 (0.89,1.01) |
|  |  |  |  | ***P*** | *P* = **2.57×10^-2^** | *P* = **4.89×10^-2^** | *P* = 9.51×10^-1^ | *P* = 9.03×10^-1^ | *P* = 3.44×10^-1^ | *P* = 4.88×10^-1^ | *P* = 6.67×10^-2^ | *P* = 7.55×10^-2^ |
| *KCNQ1* | rs2237895 | 11 | A/**C** | **OR (95%CI)** | 0.96 (0.89,1.03) | 0.99 (0.91,1.08) | 0.96 (0.89,1.03) | 0.97 (0.90,1.05) | 1.01 (0.93,1.11) | 1.04 (0.95,1.13) | 0.91 (0.85,0.98) | 0.92 (0.85,0.98) |
|  |  |  |  | ***P*** | *P* = 2.38×10^-1^ | *P* = 8.51×10^-1^ | *P* = 2.78×10^-1^ | *P* = 4.02×10^-1^ | *P* = 7.61×10^-1^ | *P* = 4.28×10^-1^ | *P* = **8.94×10^-3^** | *P* = **1.21×10^-2^** |
| *CENTD2* | rs1552224 | 11 | **T**/G | **OR (95%CI)** | 0.96 (0.86,1.08) | 0.95 (0.83,1.09) | 0.94 (0.83,1.05) | 0.93 (0.83,1.06) | 1.08 (0.94,1.23) | 1.10 (0.95,1.27) | 1.03 (0.93,1.15) | 1.03 (0.93,1.15) |
|  |  |  |  | ***P*** | *P* = 4.92×10^-1^ | *P* = 4.62×10^-1^ | *P* = 3.05×10^-1^ | *P* = 3.11×10^-1^ | *P* = 2.67×10^-1^ | *P* = 1.92×10^-1^ | *P* = 5.84×10^-1^ | *P* = 5.87×10^-1^ |
| *TSPAN8/LGR5* | rs7961581 | 12 | T/**C** | **OR (95%CI)** | 1.00 (0.92,1.08) | 0.95 (0.86,1.04) | 0.95 (0.87,1.03) | 0.94 (0.87,1.03) | 1.03 (0.94,1.13) | 1.03 (0.94,1.14) | 1.01 (0.94,1.09) | 1.01 (0.94,1.09) |
|  |  |  |  | ***P*** | *P* = 9.25×10^-1^ | *P* = 2.64×10^-1^ | *P* = 2.18×10^-1^ | *P* = 1.66×10^-1^ | *P* = 5.28×10^-1^ | *P* = 5.15×10^-1^ | *P* = 7.65×10^-1^ | *P* = 7.74×10^-1^ |
| *ZFAND6* | rs11634397 | 15 | A/**G** | **OR (95%CI)** | 1.02 (0.92,1.13) | 1.01 (0.88,1.14) | 0.99 (0.89,1.11) | 0.98 (0.88,1.10) | 1.14 (1.00,1.29) | 1.14 (1.00,1.29) | 0.89 (0.80,0.98) | 0.88 (0.80,0.98) |
|  |  |  |  | ***P*** | *P* = 7.35×10^-1^ | *P* = 9.38×10^-1^ | *P* = 8.55×10^-1^ | *P* = 7.90×10^-1^ | *P* = **4.54×10^-2^** | *P* = **4.68×10^-2^** | *P* = **2.22×10^-2^** | *P* = **1.94×10^-2^** |
| *PRC1* | rs8042680 | 15 | **A**/C | **OR (95%CI)** | 0.88 (0.70,1.11) | 0.90 (0.68,1.20) | 1.00 (0.78,1.27) | 1.02 (0.79,1.32) | 1.05 (0.79,1.39) | 1.09 (0.81,1.45) | 1.03 (0.82,1.28) | 1.03 (0.82,1.28) |
|  |  |  |  | ***P*** | *P* = 3.08×10^-1^ | *P* = 4.87×10^-1^ | *P* = 9.85×10^-1^ | *P* = 8.58×10^-1^ | *P* = 7.19×10^-1^ | *P* = 5.68×10^-1^ | *P* = 8.09×10^-1^ | *P* = 8.00×10^-1^ |
| *FTO* | rs8050136 | 16 | C/**A** | **OR (95%CI)** | 1.04 (0.95,1.14) | 0.94 (0.83,1.06) | 0.98 (0.88,1.08) | 0.94 (0.85,1.04) | 0.95 (0.85,1.06) | 0.91 (0.81,1.02) | 1.08 (0.98,1.18) | 1.06 (0.97,1.17) |
|  |  |  |  | ***P*** | *P* = 4.12×10^-1^ | *P* = 2.88×10^-1^ | *P* = 6.70×10^-1^ | *P* = 2.21×10^-1^ | *P* = 3.59×10^-1^ | *P* = 1.19×10^-1^ | *P* = 1.03×10^-1^ | *P* = 1.97×10^-1^ |
| *FTO* | rs9939609 | 16 | T/**A** | **OR (95%CI)** | 1.05 (0.95,1.15) | 0.95 (0.84,1.06) | 0.98 (0.89,1.09) | 0.94 (0.85,1.04) | 0.96 (0.85,1.07) | 0.92 (0.82,1.04) | 1.07 (0.98,1.17) | 1.06 (0.96,1.16) |
|  |  |  |  | ***P*** | *P* = 3.47×10^-1^ | *P* = 3.54×10^-1^ | *P* = 7.36×10^-1^ | *P* = 2.53×10^-1^ | *P* = 4.45×10^-1^ | *P* = 1.68×10^-1^ | *P* = 1.42×10^-1^ | *P* = 2.59×10^-1^ |
| *TCF2* | rs7501939 | 17 | C/**T** | **OR (95%CI)** | 0.95 (0.88,1.02) | 0.95 (0.87,1.03) | 1.07 (0.99,1.15) | 1.07 (0.99,1.16) | 0.97 (0.89,1.06) | 0.98 (0.90,1.06) | 0.99 (0.93,1.06) | 0.99 (0.93,1.06) |
|  |  |  |  | ***P*** | *P* = 1.23×10^-1^ | *P* = 2.25×10^-1^ | *P* = 9.83×10^-2^ | *P* = 6.95×10^-2^ | *P* = 5.00×10^-1^ | *P* = 5.73×10^-1^ | *P* = 8.34×10^-1^ | *P* = 8.65×10^-1^ |

Abbreviations: BMI, body mass index; Chr, chromosome; CI, confidence interval; HDL-C, high density lipoprotein-cholesterol; MetS, metabolic syndrome; OR, odds ratio; SNP, single nucleotide polymorphism; T2D, type 2 diabetes.

^a^ Previously reported risk alleles for T2D are shown in bold and underlined.

OR and 95% CI are indicated for the reported T2D risk allele of each SNP with MetS or MetS-related components using logistic regression under an additive assumption using the following models: model 1, age, sex and T2D status were adjusted as co-variables; and model 2, age, sex, T2D status and BMI were adjusted.

Associations with *P* value < 0.05 are shown in bold and underlined.
