# Supplementary material for: The Association of Type 2 Diabetes Loci Identified in Genome-Wide Association Studies with Metabolic Syndrome and Its Components in a Chinese Population with Type 2 Diabetes
Source: PLoS One. 2015 Nov 24;10(11):e0143607. doi: 10.1371/journal.pone.0143607 (PMC4657988; doi:10.1371/journal.pone.0143607)
Supplement: S3 Table — Abbreviations: BMI, body mass index; CI, confidence interval; DBP, diastolic blood pressure; HDL-C, high density lipoprotein-cholesterol; GRS, genotype risk score; MetS, metabolic syndrome; OR, odds ratio; Q, quartile; SBP, systolic blood pressure; T2D, type 2 diabetes; TG, triglycerides. OR and 95% CI are reported for T2D GRS quartiles with the risk for MetS components using logistic regression under an additive assumption using the following models: model 1, age and sex were adjusted as co-variables; and model 2, age, sex, and BMI were adjusted. P values are calculated for T2D GRS quartiles. P trend values are calculated for T2D GRS. All non-Gaussian distributed quantitative traits were natural logarithmically transformed to normalize distributions. a, P value calculated for T2D GRS using linear regression under an additive assumption adjusted for age and sex. b, P value calculated for T2D GRS using linear regression under an additive assumption adjusted for age, sex and BMI. Associations with P values <0.05 are shown in bold and underlined. (DOCX) [file pone.0143607.s003.docx]

**S3 Table. Non-significant associations between T2D GRS and the risk for MetS-related components in T2D patients.**

| **Quartile** | **Elevated blood pressure** | | **SBP, mmHg** | **DBP, mmHg** | **Elevated TG** | | **TG, mmol/l** | **Reduced HDL-C** | | **HDL-C, mmol/l** |
| --- | --- | --- | --- | --- | --- | --- | --- | --- | --- | --- |
|  | **( ≥ 130/85 mm Hg)** | |  |  | **( ≥ 1.7 mmol/l)** | |  | **(men: < 1.03 mmol/l;** | |  |
|  |  | |  |  |  | |  | **women: < 1.29 mmol/l)** | |  |
|  | **Model 1** | **Model 2** |  |  | **Model 1** | **Model 2** |  | **Model 1** | **Model 2** |  |
| **Q1** | 1 | 1 | 133.00 (120.00,148.00) | 80.00 ( 75.00, 90.00) | 1 | 1 | 1.64 ( 1.17, 2.46) | 1 | 1 | 1.25 ( 1.06, 1.47) |
|  |  |  |  |  |  |  |  |  |  |  |
| **Q2** | 0.91 (0.75,1.10) | 0.96 (0.79,1.17) | 130.00 (120.00,145.00) | 80.00 ( 72.50, 90.00) | 1.10 (0.93,1.30) | 1.15 (0.97,1.36) | 1.68 ( 1.17, 2.50) | 1.22 (1.03,1.46) | 1.24 (1.04,1.48) | 1.21 ( 1.03, 1.44) |
|  | *P* = 3.13×10^-1^ | *P* = 6.59×10^-1^ |  |  | *P* = 2.68×10^-1^ | *P* = 1.19×10^-1^ |  | *P* = **2.33×10^-2^** | *P* = **1.56×10^-2^** |  |
|  |  |  |  |  |  |  |  |  |  |  |
| **Q3** | 0.84 (0.69,1.01) | 0.90 (0.73,1.09) | 130.00 (120.00,145.00) | 80.00 ( 74.50, 89.00) | 1.07 (0.90,1.27) | 1.12 (0.94,1.34) | 1.66 ( 1.16, 2.42) | 1.06 (0.89,1.27) | 1.09 (0.91,1.30) | 1.23 ( 1.04, 1.44) |
|  | *P* = 6.98×10^-2^ | *P* = 2.73×10^-1^ |  |  | *P* = 4.21×10^-1^ | *P* = 1.93×10^-1^ |  | *P* = 4.93×10^-1^ | *P* = 3.72×10^-1^ |  |
|  |  |  |  |  |  |  |  |  |  |  |
| **Q4** | 0.98 (0.81,1.20) | 1.08 (0.88,1.32) | 130.00 (120.00,145.00) | 80.00 ( 75.00, 90.00) | 0.96 (0.81,1.14) | 1.02 (0.85,1.22) | 1.61 ( 1.13, 2.35) | 1.02 (0.85,1.22) | 1.05 (0.87,1.26) | 1.24 ( 1.05, 1.48) |
|  | *P* = 8.73×10^-1^ | *P* = 4.84×10^-1^ |  |  | *P* = 6.42×10^-1^ | *P* = 8.20×10^-1^ |  | *P* = 8.62×10^-1^ | *P* = 6.41×10^-1^ |  |
|  |  |  |  |  |  |  |  |  |  |  |
|  | *P*_trend_ = 7.18×10^-1^ | *P*_trend_ = 6.81×10^-1^ | *P*^a^ = 1.82×10^-1^ | *P*^a^ = 2.82×10^-1^ | *P*_trend_ = 3.36×10^-1^ | *P*_trend_ = 7.28×10^-1^ | *P*^a^ = 7.97×10^-2^ | *P*_trend_ = 6.37×10^-1^ | *P*_trend_ = 8.42×10^-1^ | *P*^a^ = 5.53×10^-1^ |
|  |  |  | *P*^b^ = 6.97×10^-1^ | *P*^b^ = 8.92×10^-1^ |  |  | *P*^b^ = 2.93×10^-1^ |  |  | *P*^b^ = 8.82×10^-1^ |

Abbreviations: BMI, body mass index; CI, confidence interval; DBP, diastolic blood pressure; HDL-C, high density lipoprotein-cholesterol; GRS, genotype risk score; MetS, metabolic syndrome; OR, odds ratio; Q, quartile; SBP, systolic blood pressure; T2D, type 2 diabetes; TG, triglycerides.

OR and 95% CI are reported for T2D GRS quartiles with the risk for MetS components using logistic regression under an additive assumption using the following models: model 1, age and sex were adjusted as co-variables; and model 2, age, sex, and BMI were adjusted. *P* values are calculated for T2D GRS quartiles. *P_trend_* values are calculated for T2D GRS.

All non-Gaussian distributed quantitative traits were natural logarithmically transformed to normalize distributions.

^a^, *P* value calculated for T2D GRS using linear regression under an additive assumption adjusted for age and sex.

^b^, *P* value calculated for T2D GRS using linear regression under an additive assumption adjusted for age, sex and BMI.

Associations with *P* values <0.05 are shown in bold and underlined.
