# Supplementary material for: The Association of Type 2 Diabetes Loci Identified in Genome-Wide Association Studies with Metabolic Syndrome and Its Components in a Chinese Population with Type 2 Diabetes
Source: PLoS One. 2015 Nov 24;10(11):e0143607. doi: 10.1371/journal.pone.0143607 (PMC4657988; doi:10.1371/journal.pone.0143607)
Supplement: S4 Table — Abbreviations: BMI, body mass index; CI, confidence interval; DBP, diastolic blood pressure; HDL-C, high density lipoprotein-cholesterol; GRS, genotype risk score; MetS, metabolic syndrome; OR, odds ratio; Q, quartile; SBP, systolic blood pressure; T2D, type 2 diabetes; TG, triglycerides; WC, waist circumference. OR and 95% CI are reported for T2D GRS quartiles with the risk for MetS components using logistic regression under an additive assumption using the following models: model 1, age, sex, and T2D status were adjusted as co-variables; and model 2, age, sex, T2D status and BMI were adjusted. P values are calculated for T2D GRS quartiles. P trend values are calculated for T2D GRS. All non-Gaussian distributed quantitative traits were natural logarithmically transformed to normalize distributions. a, P value calculated for T2D GRS using linear regression under an additive assumption adjusted for age, sex, and T2D status. b, P value calculated for T2D GRS using linear regression under an additive assumption adjusted for age, sex, T2D status and BMI. Associations with P values <0.05 are shown in bold and underlined. (DOCX) [file pone.0143607.s004.docx]

**S4 Table. Associations between T2D GRS and the risk for MetS-related components in the entire sample of cases and controls.**

| **Quartile** | **Elevated WC** | | **WC, cm** | | **Elevated blood pressure** | | **SBP, mmHg** | | **DBP, mmHg** | |
| --- | --- | --- | --- | --- | --- | --- | --- | --- | --- | --- |
|  | **(men: ≥ 90 cm;** | |  |  | **(≥ 130/85 mm Hg)** | |  |  |  |  |
|  | **women: ≥ 85 cm)** | |  |  |  | |  |  |  |  |
|  | **Model 1** | **Model 2** | **Control** | **T2D** | **Model 1** | **Model 2** | **Control** | **T2D** | **Control** | **T2D** |
| **Q1** | 1 | 1 | 79.00 (73.00, 85.00) | 89.00 (82.00, 96.00) | 1 | 1 | 116.50 (109.00, 123.00) | 133.00 (120.00,148.00) | 75.00 (70.00, 80.00) | 80.00 (75.00, 90.00) |
|  |  |  |  |  |  |  |  |  |  |  |
| **Q2** | 0.93 (0.82,1.06) | 0.97 (0.82,1.14) | 79.00 (74.00, 85.00) | 88.00 (82.00, 95.00) | 0.94 (0.82,1.08) | 0.95 (0.82,1.10) | 115.00 (107.00, 124.00) | 130.00 (120.00,145.00) | 75.00 (70.00, 80.00) | 80.00 (72.50, 90.00) |
|  | *P* = 3.04×10^-1^ | *P* = 6.95×10^-1^ |  |  | *P* = 3.50×10^-1^ | *P* = 4.78×10^-1^ |  |  |  |  |
|  |  |  |  |  |  |  |  |  |  |  |
| **Q3** | 0.88 (0.77,1.01) | 0.92 (0.78,1.09) | 79.00 (73.00, 85.00) | 88.00 (81.00, 94.00) | 0.89 (0.77,1.03) | 0.91 (0.79,1.06) | 116.00 (108.00, 123.00) | 130.00 (120.00,145.00) | 75.00 (70.00, 80.00) | 80.00 (74.50, 89.00) |
|  | *P* = 6.74×10^-2^ | *P* = 3.52×10^-1^ |  |  | *P* =1.08×10^-1^ | *P* = 2.17×10^-1^ |  |  |  |  |
|  |  |  |  |  |  |  |  |  |  |  |
| **Q4** | 0.85 (0.73,0.98) | 0.87 (0.73,1.04) | 79.00 (74.00, 85.00) | 87.00 (80.00, 94.00) | 0.99 (0.85,1.15) | 1.02 (0.87,1.19) | 115.00 (107.50, 124.00) | 130.00 (120.00,145.00) | 75.00 (70.00, 80.00) | 80.00 (75.00, 90.00) |
|  | *P* = **2.14×10^-2^** | *P* = 1.32×10^-1^ |  |  | *P* = 9.13×10^-1^ | *P* = 8.10×10^-1^ |  |  |  |  |
|  |  |  |  |  |  |  |  |  |  |  |
|  | *P*_trend_ =7.66×10^-2^ | *P*_trend_ = 2.44×10^-1^ | *P*^a^ = 8.28×10^-2^ | | *P*_trend_ = 6.29×10^-1^ | *P*_trend_ = 8.21×10^-1^ | *P*^a^ = 1.80×10^-1^ | | *P*^a^ = 3.42×10^-1^ | |
|  |  |  | *P*^b^ = 2.19×10^-1^ | |  |  | *P*^b^ = 2.81×10^-1^ | | *P*^b^ = 5.27×10^-1^ | |
|  |  |  |  |  |  |  |  |  |  |  |
| **Quartile** | **Elevated triglycerides** | | **TG, mmol/l** | | **Reduced HDL-C** | | **HDL-C, mmol/l** | |  |  |
|  | **(≥ 1.7 mmol/l)** | |  |  | **(men: < 1.03 mmol/l;** | |  |  |  |  |
|  |  | |  |  | **women: < 1.29 mmol/l)** | |  |  |  |  |
|  | **Model 1** | **Model 2** | **Control** | **T2D** | **Model 1** | **Model 2** | **Control** | **T2D** |  |  |
| **Q1** | 1 | 1 | 1.03 (0.80, 1.29) | 1.64 (1.17, 2.46) | 1 | 1 | 1.38 (1.17, 1.61) | 1.25 (1.06, 1.47) |  |  |
|  |  |  |  |  |  |  |  |  |  |  |
| **Q2** | 1.10 (0.93,1.30) | 1.14 (0.97,1.35) | 1.01 (0.77, 1.27) | 1.68 (1.17, 2.50) | 1.14 (1.02,1.29) | 1.14 (1.01,1.30) | 1.37 (1.15, 1.61) | 1.21 (1.03, 1.44) |  |  |
|  | *P* = 2.57×10^-1^ | *P* = 1.18×10^-1^ |  |  | *P* = **4.71×10^-2^** | *P* = **3.70×10^-2^** |  |  |  |  |
|  |  |  |  |  |  |  |  |  |  |  |
| **Q3** | 1.05 (0.89,1.24) | 1.09 (0.92,1.30) | 1.01 (0.77, 1.30) | 1.66 (1.16, 2.42) | 1.03 (0.91,1.18) | 1.04 (0.91,1.19) | 1.38 (1.16, 1.60) | 1.23 (1.04, 1.44) |  |  |
|  | *P* = 5.78×10^-1^ | *P* = 3.14×10^-1^ |  |  | *P* = 6.37×10^-1^ | *P* =5.38×10^-1^ |  |  |  |  |
|  |  |  |  |  |  |  |  |  |  |  |
| **Q4** | 0.95 (0.80,1.13) | 1.01 (0.85,1.21) | 1.02 (0.76, 1.33) | 1.61 (1.13, 2.35) | 1.04 (0.91,1.20) | 1.05 (0.92,1.21) | 1.37 (1.18, 1.64) | 1.24 (1.05, 1.48) |  |  |
|  | *P* = 5.88×10^-1^ | *P* = 9.05×10^-1^ |  |  | *P* = 5.42×10^-1^ | *P* = 4.67×10^-1^ |  |  |  |  |
|  |  |  |  |  |  |  |  |  |  |  |
|  | *P*_trend_ = 3.24×10^-1^ | *P*_trend_ = 6.85×10^-1^ | *P*^a^ = 7.96×10^-2^ | | *P*_trend_ = 7.83×10^-1^ | *P*_trend_ = 8.44×10^-1^ | *P*^a^ = 7.45×10^-1^ | |  |  |
|  |  |  | *P*^b^ = 1.11×10^-1^ | |  |  | *P*^b^ = 9.03×10^-1^ | |  |  |

Abbreviations: BMI, body mass index; CI, confidence interval; DBP, diastolic blood pressure; HDL-C, high density lipoprotein-cholesterol; GRS, genotype risk score; MetS, metabolic syndrome; OR, odds ratio; Q, quartile; SBP, systolic blood pressure; T2D, type 2 diabetes; TG, triglycerides; WC, waist circumference.

OR and 95% CI are reported for T2D GRS quartiles with the risk for MetS components using logistic regression under an additive assumption using the following models: model 1, age, sex, and T2D status were adjusted as co-variables; and model 2, age, sex, T2D status and BMI were adjusted. *P* values are calculated for T2D GRS quartiles. *P_trend_* values are calculated for T2D GRS.

All non-Gaussian distributed quantitative traits were natural logarithmically transformed to normalize distributions.

^a^, *P* value calculated for T2D GRS using linear regression under an additive assumption adjusted for age, sex, and T2D status.

^b^, *P* value calculated for T2D GRS using linear regression under an additive assumption adjusted for age, sex, T2D status and BMI.

Associations with *P* values <0.05 are shown in bold and underlined.
