# Supplementary material for: The Association of Type 2 Diabetes Loci Identified in Genome-Wide Association Studies with Metabolic Syndrome and Its Components in a Chinese Population with Type 2 Diabetes
Source: PLoS One. 2015 Nov 24;10(11):e0143607. doi: 10.1371/journal.pone.0143607 (PMC4657988; doi:10.1371/journal.pone.0143607)
Supplement: S5 Table — Abbreviations: BMI, body mass index; Chr, chromosome; CI, confidence interval; DMS, Chinese National Diabetes and Metabolic Disorders Study; MetS, metabolic syndrome; OR, odds ratio; SNP, single nucleotide polymorphism; T2D, type 2 diabetes. a Previously reported risk alleles for T2D are shown in bold and underlined. OR and 95% CI are indicated for the reported T2D risk allele of each SNP using logistic regression under an additive assumption using the following models: model 1, age and sex were adjusted as co-variables; and model 2, age, sex, and BMI were adjusted. Associations with P values < 0.05 are shown in bold and underlined. (DOCX) [file pone.0143607.s005.docx]

**S5 Table. Associations between SNPs with MetS T2D and non-MetS T2D compared with non-MetS super controls in DMS.**

| **Gene** | **SNP** | **Chr.** | **Major/minor allele^a^** |  | **Non-MetS T2D vs Non-MetS super controls** | | **MetS T2D vs Non-MetS super controls** | |
| --- | --- | --- | --- | --- | --- | --- | --- | --- |
|  |  |  |  |  | **(1,518:1,956)** | | **(3,651:1,956)** | |
|  |  |  |  |  | **Model 1** | **Model 2** | **Model 1** | **Model 2** |
| *NOTCH2* | rs10923931 | 1 | G/**T** | **OR (95%CI)** | 0.93 (0.72,1.20) | 0.94 (0.73,1.23) | 0.95 (0.77,1.17) | 0.93 (0.70,1.23) |
|  |  |  |  | ***P*** | *P* = 5.81×10^-1^ | *P* = 6.62×10^-1^ | *P* = 6.28×10^-1^ | *P* = 5.98×10^-1^ |
| *BCL11A* | rs243021 | 2 | **T**/C | **OR (95%CI)** | 1.15 (1.04,1.28) | 1.15 (1.03,1.28) | 1.06 (0.97,1.16) | 1.02 (0.92,1.15) |
|  |  |  |  | ***P*** | *P* = **7.91×10^-3^** | *P* = **1.52×10^-2^** | *P* = 1.80×10^-1^ | *P* = 6.87×10^-1^ |
| *GCKR* | rs780094 | 2 | A/**G** | **OR (95%CI)** | 1.09 (0.99,1.20) | 1.09 (0.99,1.20) | 1.00 (0.92,1.08) | 0.98 (0.88,1.08) |
|  |  |  |  | ***P*** | *P* = 7.74×10^-2^ | *P* = 9.42×10^-2^ | *P* = 9.41×10^-1^ | *P* = 6.46×10^-1^ |
| *PPARG* | rs1801282 | 3 | **C**/G | **OR (95%CI)** | 1.18 (0.96,1.45) | 1.19 (0.97,1.47) | 1.04 (0.88,1.22) | 1.09 (0.88,1.35) |
|  |  |  |  | ***P*** | *P* = 1.05×10^-1^ | *P* = 9.93×10^-2^ | *P* = 6.62×10^-1^ | *P* = 4.39×10^-1^ |
| *ADAMTS9* | rs4607103 | 3 | **C**/T | **OR (95%CI)** | 1.06 (0.96,1.18) | 1.08 (0.96,1.19) | 0.99 (0.91,1.08) | 0.99 (0.88,1.11) |
|  |  |  |  | ***P*** | *P* = 2.05×10^-1^ | *P* = 1.91×10^-1^ | *P* = 7.99×10^-1^ | *P* = 8.75×10^-1^ |
| *WFS1* | rs10010131 | 4 | **G**/A | **OR (95%CI)** | 1.20 (0.95,1.52) | 1.27 (1.00,1.61) | 1.33 (1.10,1.64) | 1.41 (1.09,1.82) |
|  |  |  |  | ***P*** | *P* = 1.27×10^-1^ | *P* = 5.40×10^-2^ | *P* = **4.17×10^-3^** | *P* = **1.01×10^-2^** |
| *ZBED3* | rs4457053 | 5 | A/**G** | **OR (95%CI)** | 0.87 (0.69,1.10) | 0.88 (0.69,1.12) | 1.02 (0.85,1.23) | 1.01 (0.79,1.30) |
|  |  |  |  | ***P*** | *P* = 2.37×10^-1^ | *P* = 2.85×10^-1^ | *P* = 8.14×10^-1^ | *P* = 9.10×10^-1^ |
| *CDKAL1* | rs7756992 | 6 | **G**/A | **OR (95%CI)** | 1.22 (1.10,1.33) | 1.22 (1.10,1.35) | 1.18 (1.09,1.28) | 1.27 (1.14,1.41) |
|  |  |  |  | ***P*** | *P* = **1.16×10^-4^** | *P* = **1.14×10^-4^** | *P* = **6.01×10^-5^** | *P* = **1.44×10^-5^** |
| *JAZF1* | rs864745 | 7 | **A**/G | **OR (95%CI)** | 0.98 (0.88,1.10) | 0.98 (0.88,1.11) | 0.98 (0.89,1.09) | 1.00 (0.88,1.14) |
|  |  |  |  | ***P*** | *P* = 7.57×10^-1^ | *P* = 7.96×10^-1^ | *P* = 7.68×10^-1^ | *P* = 9.93×10^-1^ |
| *KLF14* | rs972283 | 7 | **G**/A | **OR (95%CI)** | 0.89 (0.80,0.99) | 0.88 (0.79,0.99) | 1.02 (0.93,1.11) | 1.05 (0.93,1.18) |
|  |  |  |  | ***P*** | *P* = **3.81×10^-2^** | *P* = **3.27×10^-2^** | *P* = 6.94×10^-1^ | *P* = 4.39×10^-1^ |
| *TP53INP1* | rs896854 | 8 | G/**A** | **OR (95%CI)** | 1.03 (0.93,1.15) | 1.03 (0.93,1.15) | 1.05 (0.97,1.15) | 1.06 (0.95,1.19) |
|  |  |  |  | ***P*** | *P* = 5.54×10^-1^ | *P* = 5.74×10^-1^ | *P* = 2.37×10^-1^ | *P* = 2.99×10^-1^ |
| *CDKN2BAS* | rs10811661 | 9 | **T**/C | **OR (95%CI)** | 1.23 (1.12,1.37) | 1.22 (1.11,1.35) | 1.19 (1.10,1.30) | 1.27 (1.14,1.41) |
|  |  |  |  | ***P*** | *P* = **2.15×10^-5^** | *P* = **9.47×10^-5^** | *P* = **3.51×10^-5^** | *P* = **1.95×10^-5^** |
| *CHCHD9* | rs13292136 | 9 | **C**/T | **OR (95%CI)** | 1.03 (0.88,1.22) | 1.01 (0.85,1.19) | 0.98 (0.85,1.12) | 0.99 (0.83,1.19) |
|  |  |  |  | ***P*** | *P* = 7.01×10^-1^ | *P* = 9.37×10^-1^ | *P* = 7.58×10^-1^ | *P* = 9.15×10^-1^ |
| *TCF7L2* | rs7903146 | 10 | C/**T** | **OR (95%CI)** | 1.43 (1.14,1.79) | 1.42 (1.13,1.79) | 1.16 (0.94,1.42) | 1.04 (0.80,1.35) |
|  |  |  |  | ***P*** | *P* = **2.08×10^-3^** | *P* = **2.96×10^-3^** | *P* = 1.59×10^-1^ | *P* = 7.70×10^-1^ |
| *CDC123/CAMK1D* | rs12779790 | 10 | A/**G** | **OR (95%CI)** | 1.14 (1.00,1.29) | 1.14 (1.00,1.30) | 1.01 (0.91,1.13) | 1.11 (0.97,1.28) |
|  |  |  |  | ***P*** | *P* = 5.06×10^-1^ | *P* = **4.63×10^-2^** | *P* = 8.14×10^-1^ | *P* = 1.37×10^-1^ |
| *HHEX* | rs1111875 | 10 | A/**G** | **OR (95%CI)** | 1.11 (0.99,1.231 | 1.10 (0.99,1.23) | 1.09 (0.99,1.19) | 1.06 (0.94,1.19) |
|  |  |  |  | ***P*** | *P* = 6.65×10^-2^ | *P* = 7.73×10^-2^ | *P* = 6.93×10^-2^ | *P* = 3.46×10^-1^ |
| *MTNRIB* | rs10830963 | 11 | C/**G** | **OR (95%CI)** | 1.08 (0.98,1.20) | 1.07 (0.96,1.18) | 1.03 (0.94,1.11) | 1.03 (0.92,1.15) |
|  |  |  |  | ***P*** | *P* = 1.17×10^-1^ | *P* = 2.07×10^-1^ | *P* = 5.66×10^-1^ | *P* = 5.91×10^-1^ |
| *KCNQ1* | rs2237895 | 11 | A/**C** | **OR (95%CI)** | 1.31 (1.18,1.46) | 1.30 (1.16,1.45) | 1.22 (1.12,1.34) | 1.28 (1.14,1.44) |
|  |  |  |  | ***P*** | *P* = **1.13×10^-6^** | *P* = **5.20×10^-6^** | *P* = **2.07×10^-5^** | *P* = **5.57×10^-5^** |
| *CENTD2* | rs1552224 | 11 | **T**/G | **OR (95%CI)** | 1.03 (0.86,1.22) | 1.01 (0.85,1.20) | 1.02 (0.88,1.18) | 0.97 (0.81,1.18) |
|  |  |  |  | ***P*** | *P* = 7.58×10^-1^ | *P* = 8.75×10^-1^ | *P* = 7.77×10^-1^ | *P* = 7.94×10^-1^ |
| *TSPAN8/LGR5* | rs7961581 | 12 | T/**C** | **OR (95%CI)** | 1.07 (0.95,1.21) | 1.05 (0.92,1.19) | 1.10 (1.00,1.22) | 1.06 (0.93,1.21) |
|  |  |  |  | ***P*** | *P* = 2.79×10^-1^ | *P* = 4.73×10^-1^ | *P* = 6.23×10^-2^ | *P* = 3.86×10^-1^ |
| *ZFAND6* | rs11634397 | 15 | A/**G** | **OR (95%CI)** | 1.05 (0.90,1.23) | 1.06 (0.90,1.25) | 1.00 (0.88,1.15) | 0.97 (0.82,1.16) |
|  |  |  |  | ***P*** | *P* = 5.26×10^-1^ | *P* = 4.63×10^-1^ | *P* = 9.70×10^-1^ | *P* = 7.59×10^-1^ |
| *PRC1* | rs8042680 | 15 | **A**/C | **OR (95%CI)** | 0.84 (0.60,1.19) | 0.87 (0.61,1.23) | 1.02 (0.75,1.39) | 1.16 (0.76,1.75) |
|  |  |  |  | ***P*** | *P* = 3.35×10^-1^ | *P* = 4.35×10^-1^ | *P* = 9.04×10^-1^ | *P* = 4.79×10^-1^ |
| *FTO* | rs8050136 | 16 | C/**A** | **OR (95%CI)** | 1.09 (0.94,1.27) | 1.07 (0.91,1.24) | 1.20 (1.06,1.36) | 1.07 (0.91,1.25) |
|  |  |  |  | ***P*** | *P* = 2.41×10^-1^ | *P* = 4.19×10^-1^ | *P* = **5.14×10^-3^** | *P* = 4.44×10^-1^ |
| *FTO* | rs9939609 | 16 | T/**A** | **OR (95%CI)** | 1.11 (0.96,1.29) | 1.09 (0.93,1.27) | 1.22 (1.08,1.38) | 1.09 (0.92,1.28) |
|  |  |  |  | ***P*** | *P* = 1.56×10^-1^ | *P* = 2.84×10^-1^ | *P* = **2.05×10^-3^** | *P* = 3.07×10^-1^ |
| *TCF2* | rs7501939 | 17 | C/**T** | **OR (95%CI)** | 1.08 (0.97,1.20) | 1.07 (0.96,1.20) | 1.13 (1.03,1.24) | 1.12 (1.00,1.26) |
|  |  |  |  | ***P*** | *P* = 1.60×10^-1^ | *P* = 2.13×10^-1^ | *P* = **1.04×10^-2^** | *P* = 5.53×10^-2^ |

Abbreviations: BMI, body mass index; Chr, chromosome; CI, confidence interval; DMS, Chinese National Diabetes and Metabolic Disorders Study; MetS, metabolic syndrome; OR, odds ratio; SNP, single nucleotide polymorphism; T2D, type 2 diabetes.

^a^ Previously reported risk alleles for T2D are shown in bold and underlined.

OR and 95% CI are indicated for the reported T2D risk allele of each SNP using logistic regression under an additive assumption using the following models: model 1, age and sex were adjusted as co-variables; and model 2, age, sex, and BMI were adjusted.

Associations with *P* values < 0.05 are shown in bold and underlined.
